# Targeted modification of the *Per2* clock gene alters circadian function in *mPer2*^*luciferase*^ (*mPer2*^*Luc*^) mice

**DOI:** 10.1371/journal.pcbi.1008987

**Published:** 2021-05-28

**Authors:** Martin R. Ralph, Shu-qun Shi, Carl H. Johnson, Pavel Houdek, Tenjin C. Shrestha, Priya Crosby, John S. O’Neill, Martin Sládek, Adam R. Stinchcombe, Alena Sumová

**Affiliations:** 1 Department of Psychology, University of Toronto, Toronto, Ontario, Canada; 2 Department of Biological Sciences, Vanderbilt University, Nashville, Tennessee, United States of America; 3 Laboratory of Biological Rhythms, Institute of Physiology, the Czech Academy of Sciences, Prague, Czech Republic; 4 Department of Cell and Systems Biology, University of Toronto, Toronto, Ontario, Canada; 5 MRC Laboratory of Molecular Biology, Cambridge, United Kingdom; 6 Department of Mathematics, University of Toronto, Toronto, Ontario, Canada; University of California Los Angeles, UNITED STATES

## Abstract

Modification of the *Per2* clock gene in *mPer2*^*Luc*^ reporter mice significantly alters circadian function. Behavioral period in constant dark is lengthened, and dissociates into two distinct components in constant light. Rhythms exhibit increased bimodality, enhanced phase resetting to light pulses, and altered entrainment to scheduled feeding. Mechanistic mathematical modelling predicts that enhanced protein interactions with the modified mPER2 C-terminus, combined with differential clock regulation among SCN subregions, can account for effects on circadian behavior via increased *Per2* transcript and protein stability. PER2::LUC produces greater suppression of CLOCK:BMAL1 E-box activity than PER2. *mPer2*^*Luc*^ carries a 72 bp deletion in exon 23 of *Per2*, and retains a neomycin resistance cassette that affects rhythm amplitude but not period. The results show that *mPer2*^*Luc*^ acts as a circadian clock mutation illustrating a need for detailed assessment of potential impacts of c-terminal tags in genetically modified animal models.

## Author summary

Engineered genes that are modified to express bioluminescent signals can be used to monitor cellular processes by reflecting the actual state of the process in real time. For circadian rhythm studies, a widely used reporter model (*mPer2*^*Luc*^) expresses firefly luciferase under the control of the clock gene *Period 2* (*Per2*), encoding a fusion protein (PER2::LUCIFERASE) which produces rhythms of bioluminescence. We present evidence that the engineered protein produces extensive changes in iconic circadian behavioral patterns in mice. The evidence comes from analysis of long-term recordings of circadian behavior in *mPer2*^*Luc*^ mice in constant darkness and constant light, together with responsiveness to light pulses and entrainment to restricted food availability. By integrating a well-established mathematical model of circadian rhythm generation in the suprachiasmatic nuclei with the known functional heterogeneity within the nucleus, we recapitulated the observed locomotor behavioral patterns in WT and *mPer2*^*Luc*^ mice. Functional alterations to the stability of PER2 protein predicted from the model were substantiated by increased repression strength of PER2::LUC over WT PER2 on CLOCK:BMAL1 transcriptional activation. Therefore, *mPer2*^*Luc*^ is a clock mutation that provides new insights into the functional neuroanatomy of the mammalian circadian system and the critical importance of *Per2* regulation in rhythm generation.

## Introduction

A commonly used and highly efficient approach for monitoring cellular processes is to engineer genes encoding critical components of the process, so that they create benign products which signal accurately the natural activity of that gene. A reliable report of the state of any process requires first that the engineered product mirrors the abundance of the native product, and second, that it has no relevant effect on the process itself. In circadian rhythm studies, an ideal reporter should reproduce the phase, amplitude, and period of the organism’s biological clock without affecting the operation of the timing mechanism itself. In the mouse, a widely used circadian reporter (*mPer2*^*Luc*^) expresses firefly luciferase regulated by the clock gene *Period 2* (*Per2*) [1]. The engineered gene encodes a fusion protein (PER2::LUCIFERASE, PER2::LUC) which in the presence of the substrate, luciferin, produces rhythms of bioluminescence. The signal is considered to be a high-fidelity reflection of newly translated PER2 in recordings from organotypic tissue culture, freely moving animals, and cultured cells [1–5].

As a core component of the mammalian molecular clock, the rhythmic transcription of *Per2* is driven by the positive transcription factor CLOCK:BMAL1. The workings of the molecular clock, and the role of *Per2*, have been reviewed extensively [6, 7]. Following translation, PER2 accumulates in the cytoplasm where it interacts with other clock proteins, specifically cryptochromes (CRYs). PER2/CRY heterodimers are translocated into the nucleus where they repress the activity of CLOCK:BMAL1, thereby completing a circadian transcription/translation feedback loop (TTFL). Post-translational modifications of PER2 regulate its stability as well as dynamics of PER2/CRY nuclear-cytoplasmatic shuttling, both of which contribute to the precise control of the circadian period [8–12]. Recent studies indicate that both transcription [13–17] and translation [18] of *Per2* respond directly to sensory stimulation so that PER2::LUC bioluminescence reflects a combination of circadian phase along with direct real time sensing of ambient conditions.

In a study of 12 inbred strains of mice, Schwartz & Zimmerman (1990) found that free-running period of the locomotor activity in constant dark (DD) ranged from 22.93 h to 23.94 h [19]. The C57Bl6 strain used to generate the *mPer2*^*Luc*^ mouse was reported to have a mean period of about 23.77 h [1, 4]. Therefore genetic variability, age, and laboratory conditions (e.g., access to an activity wheel) can contribute to period variance in free-running behavioral rhythms [20–22]. In contrast the *ex vivo* period of bioluminescence rhythms reported from the SCN ranges from 23.5 h to 26.5 h and exceeds the reported range for *in vivo* behavioral rhythms [1, 10, 19, 23–25]. Slightly longer periods compared with widely reported mouse behavior, have been seen in SCN explants using PER1:LUC [12] and CRY1-LUC [26] reporters. Damage to tissue during preparation of the organotypic explant, or the chemical microenvironments of culture media seem to be unlikely sources of period alteration. Transplants of SCN tissue or dispersed cells, produce rhythms within the ranges expected from the intact donor genotypes [27–31]. However, systematic comparisons of these influences on period in organotypic culture have not been reported to our knowledge.

Here we have tested an alternative hypothesis–that properties of the circadian molecular clock are altered by the fusion of the LUC moiety to PER2. The rationale for the hypothesis is based on our earlier report that circadian period of behavioral rhythms in constant light (LL), which is generally longer than 24 h in C57Bl6 mice, is significantly shorter than 24 h in *mPer2*^*Luc*^ mice [25]. To test this hypothesis, we conducted a broad longitudinal analysis of the circadian behavioral activity of *mPer2*^*Luc*^ mice in LL and constant dark (DD), along with entrainment to light pulses and restricted food availability, concluding that the gene modification produces extensive changes in characteristic circadian regulation of behavior. Then, using well-established mathematical models of mammalian circadian rhythm generation in the SCN [32–35], we recapitulated observed locomotor behavioral patterns, and identified processes linked to altered *Per2* gene expression that can account for the altered behaviors in *mPer2*^*Luc*^ mice. Potential mechanisms based on predictions from the models were substantiated by testing repression strengths of PER2::LUC versus WT PER2 on CLOCK:BMAL1 transcriptional activation. Finally, we suggest a neuroanatomical mechanism to explain the complex rhythmic expression that is found in the behavior of *mPer2*^*Luc*^ mice in DD and LL.

## Results

### Period of circadian locomotor activity rhythms of *mPer2*^*Luc/Luc*^ mice lengthens in DD

**Fig 1:**
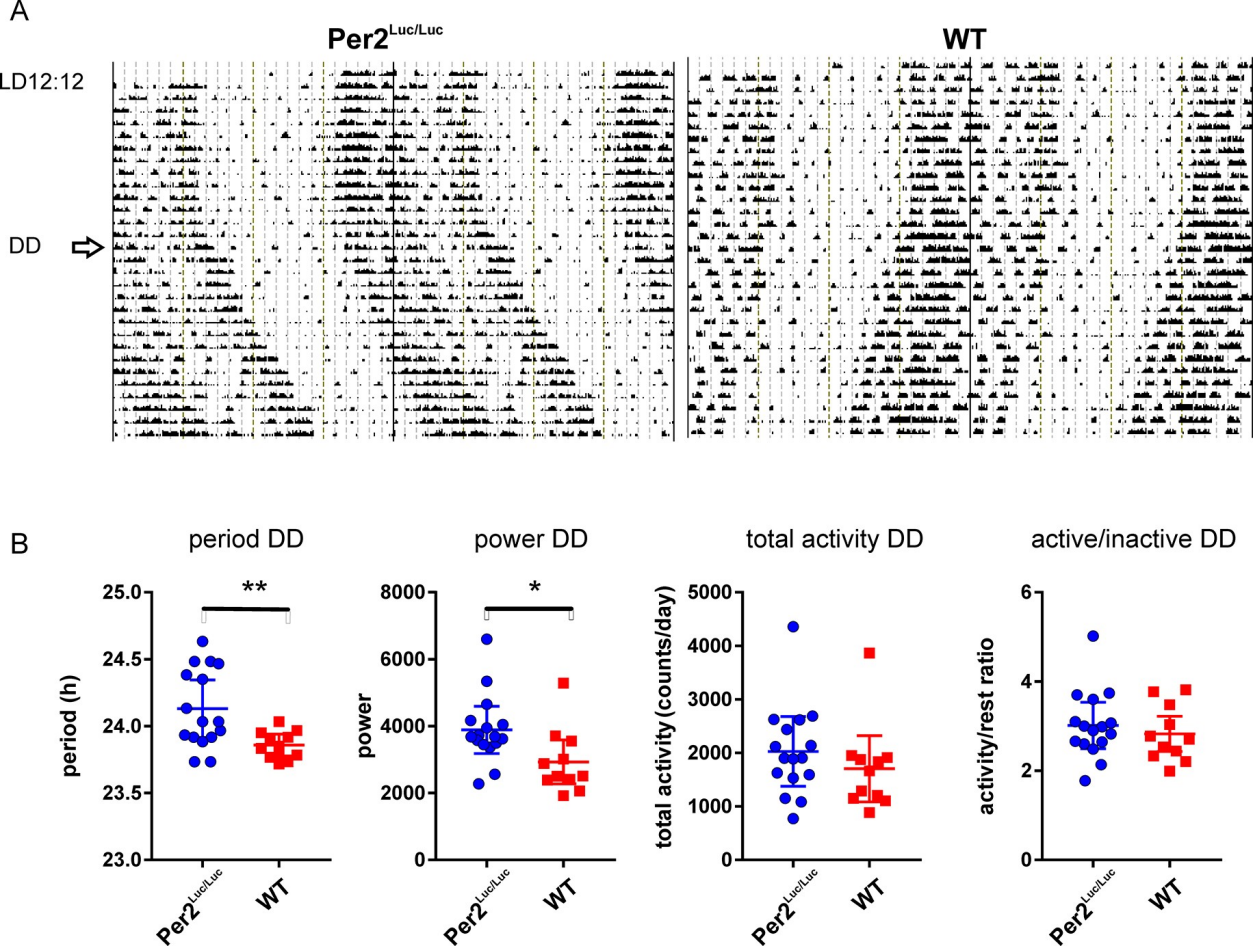
Genotype differences in OFA rhythms in DD. **A**. Representative activity records from one m*Per2*^*uc/Luc*^ and one WT C57Bl6 control animals maintained in LD and released into DD (arrow). **B**. Comparison of behavioral characteristics of m*Per2*^*uc/Luc*^ (n = 16) and WT (n = 11) in DD. Significantly longer period and power of the *mPer2*^*Luc/Luc*^ rhythms in DD. No difference in total amount of activity nor amplitude of the rhythm (activity/rest ratio). (* *P*<0.05; ** *P*<0.01). Additional records are shown in **S1A–S1C Fig**.

**Fig 2:**
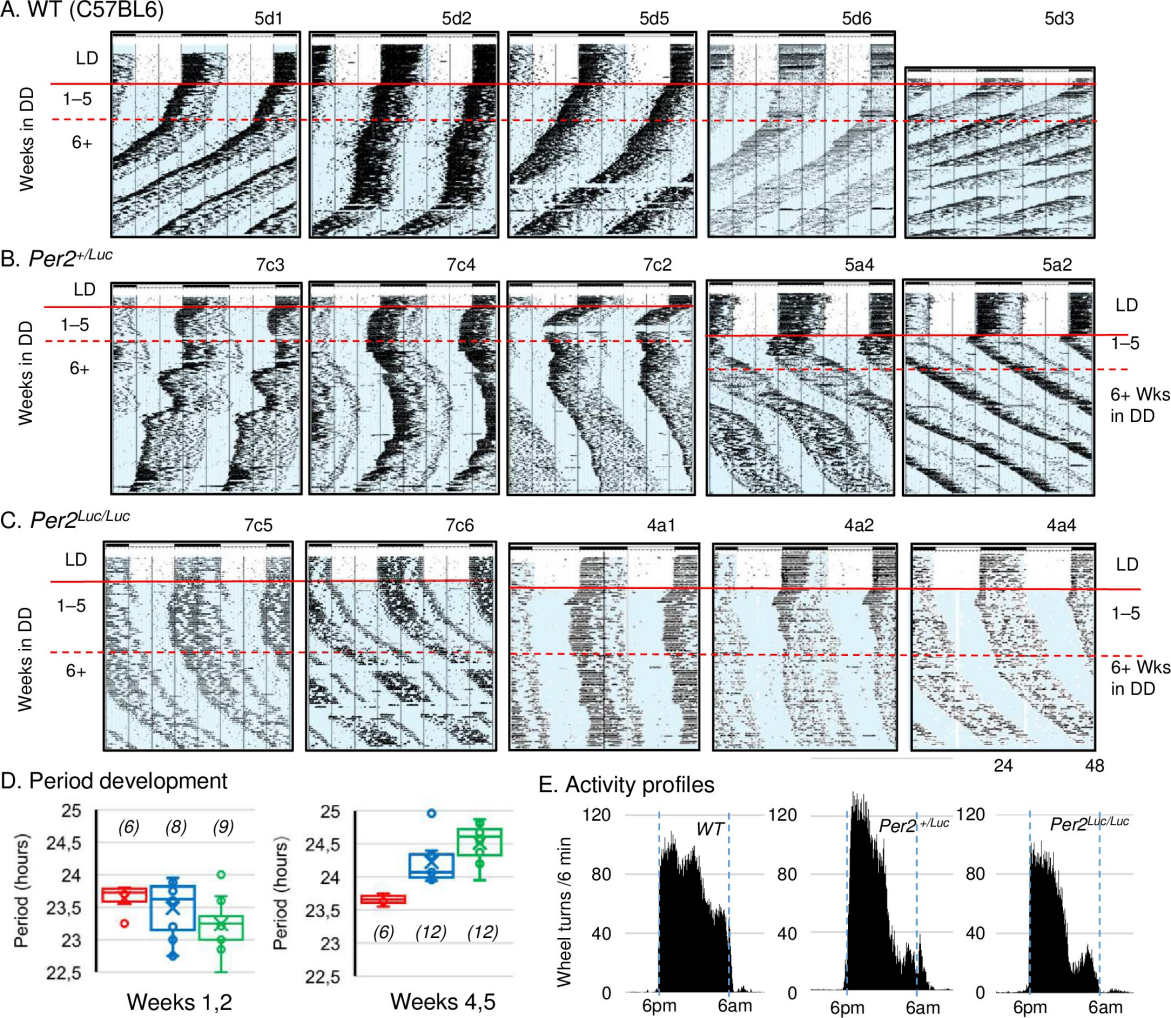
Genotype differences in RWA rhythms in DD. **A, B, C.** Representative long term records of activity from several experiments from WT C57BL6, *mPer2*^+*/Luc*^, and *mPer2*^*Luc/Luc*^ mice, respectively. All animals were entrained to LD12:12 prior release into DD. WT animals **(A)** show the expected range of periods (<24 h in DD). Homozygous *mPer2*^*Luc/Luc*^ mice **(C)** exhibit long period (>24 h in DD); and heterozygous *Per2*^*+/Luc*^ mice **(B)** exhibit individual variance with patterns ranging between the wild type and mutant characteristics, as well as period instability over time in DD. **D.** Average periods of the 3 genotypes over the 4–10 days following transition from LD to DD (left) and days 25–35 in DD (right). Cumulative data from several experiments. N/group is shown in parentheses all other comparisons = *ns*. The region analyzed is indicated by horizontal red lines on the actograms. **E.** Activity profiles for WT, *mPer2*^*+/Luc*^ and *mPer2*^*Luc/Luc*^ mice in LD12:12. Dark half of the cycle is indicated by blue rectangles with lights off from 6pm to 6 am. The grand average from 6 animals per genotype was produced from individual averages of the last 10 days in LD prior to transfer to constant conditions as illustrated in **A, B, C**.

Two methods were used to analyze the effect of PER2::LUC on circadian locomotor behavior at two locations. In the Prague facility, behavior was recorded as spontaneous open field activity (OFA) using infrared activity detectors, and in the Toronto facility as goal directed behavior using running wheel activity (RWA). Homozygous (*mPer2*^*Luc*/*Luc*^) and C57Bl6 wild type (WT) control mice were maintained under LD12:12 to establish entrainment to a common LD cycle, then were released into DD for several weeks. Significant genotype differences in the period of free-running rhythms were exhibited in both OFA **(Fig 1)** and RWA **(Fig 2)** recording environments.

In open field conditions, immediately upon releasing into DD, *mPer2*^*Luc/Luc*^ mice exhibited a free-running rhythm with a significantly longer period than control mice (WT: 23.86 ± 0.03, n = 11; *mPer2*^*Luc/Luc*^: 24.13 ± 0.07, n = 16; *P* = .0028) **(Fig 1A)**. Rhythm amplitude (i.e. power as determined by the chi-squared periodogram) was higher in *mPer2*^*Luc/Luc*^ mice (*P* = .0212), suggesting a strengthening of the circadian regulation of the output rhythm. Total activity and activity/inactivity (*α/ρ*) ratios were not different between the two strains (*P* = .3291 and .4871, respectively) (**Fig 1B**). Additional examples of activity records are shown in **S1–S3 Figs.**

In RWA recordings, the genotype differences in steady state period took longer to appear compared with OFA. Average periods during the first 2 weeks in DD were all <24 h **(Fig 2A, 2B and 2C)**, and the average period for *mPer2*^*Luc/Luc*^ was slightly shorter than for WT control mice (WT: 23.64 ± 0.09, *n* = 6; *mPer2*^*Luc/Luc*^: 23.24 ± 0.12, *n* = 12) **(Fig 2D**, *left graph***)**. *mPer2*^*Luc/Luc*^ period lengthened over weeks 2–5 (**Fig 2D**, *right graph*). Thereafter, the WT period remained <24 h for the duration of the experiment in DD (for examples see **Fig 2B**); whereas in *mPer2*^*Luc/Luc*^ mice it consistently lengthened, so that when assessed after 4 weeks in DD, the period was longer in *mPer2*^*Luc*^ animals (WT: 23.66 ± 0.03, *n* = 6; *mPer2*^*Luc/Luc*^, 24.51 ± 0.12, *n* = 12) **(Fig 2C and 2D)**. Therefore, using either OFA or RWA, the WT mouse retains a <24 h circadian period in DD and the *mPer2*^*Luc/Luc*^ animals produce a period >24 h.

The RWA recorded periods of heterozygous *mPer2*^*+/Luc*^ animals from a WT x *mPer2*^*Luc/Luc*^ cross exhibited a range between WT and *mPer2*^*Luc/Luc*^ (*mPer2*^*+/Luc*^: 23.49 ± 0.13, *n* = 12) **(Fig 2B)**. Some animals produced records that were similar in basic characteristics to either WT or *mPer2*^*Luc/Luc*^. Periods tended to be unstable, occasionally switching between long and short unpredictably. Average period over several months for *mPer2*^*+/Luc*^ mice was intermediate between WT and *mPer2*^*Luc/Luc*^ genotypes **(Fig 2D)**. The heterozygote phenotype may be more accurately characterized by the instability and variance of period in long-term individual records than by an average period for the genotype.

One-way ANOVA with Tukey’s *post hoc* test revealed no statistically significant period differences among genotypes in the first 2 weeks in DD (*F* = 2.444802; *P* = 0.105718; N = 6 WT, 12 *mPer2*^*+/Luc*^ and 12 *mPer2*^*Luc/Luc*^), although t-test with Welch’s correction showed a marginally significant difference (*P* = 0.0344) between WT and *mPer2*^*Luc*^. Significant differences were found in Weeks 4–5 (*F* = 15.888077; *P* = 0.000074) with the difference between WT (N = 6) and *mPer2*^*+/Luc*^ (N = 8) at *P* = 0.0001 and between WT and *mPer2*^*Luc/Luc*^ (N = 9) at *P* = 0.0031.

Because the periods for WT and *mPer2*^*Luc/Luc*^ in DD are consistently <24 h and >24 h, respectively, entrainment characteristics in LD might be altered. To examine this, RWA patterns in the final 10 days in LD12:12 were assessed for the 6 animals from each genotype with the highest levels of activity. The RWA group averages (grand averages) for every 6 minutes in the dark interval are shown in **Fig 2E**. The results show qualitatively different patterns among the three genotypes. Although visual examination of the records indicates an increased bimodal expression of activity in mutants versus WT, quantitative analysis of bimodality *per se* is not appropriate using RWA in this case (see Discussion). Nonetheless, a 2-way ANOVA comparing activity among the 3 h nighttime quartiles showed main effects of genotype (*F*_*G*_ = 8.127; P = 0.0008) and time (*F*_*T*_ *=* 36.5779; P < 0.00001). *Post hoc* tests (Scheffé, multiple t-tests) indicate a significantly lower amount of activity from *mPer2*^*Luc/Luc*^ mice compared with WT during the 3^rd^ (P = 0.000125) and 4^th^ (P = 0.04301448) quartiles, accounting for the increased appearance of bimodality.

### *mPer2*^*Luc/Luc*^ mice exhibit two dissociated activity rhythms in LL with a predominant short period component

**Fig 3:**
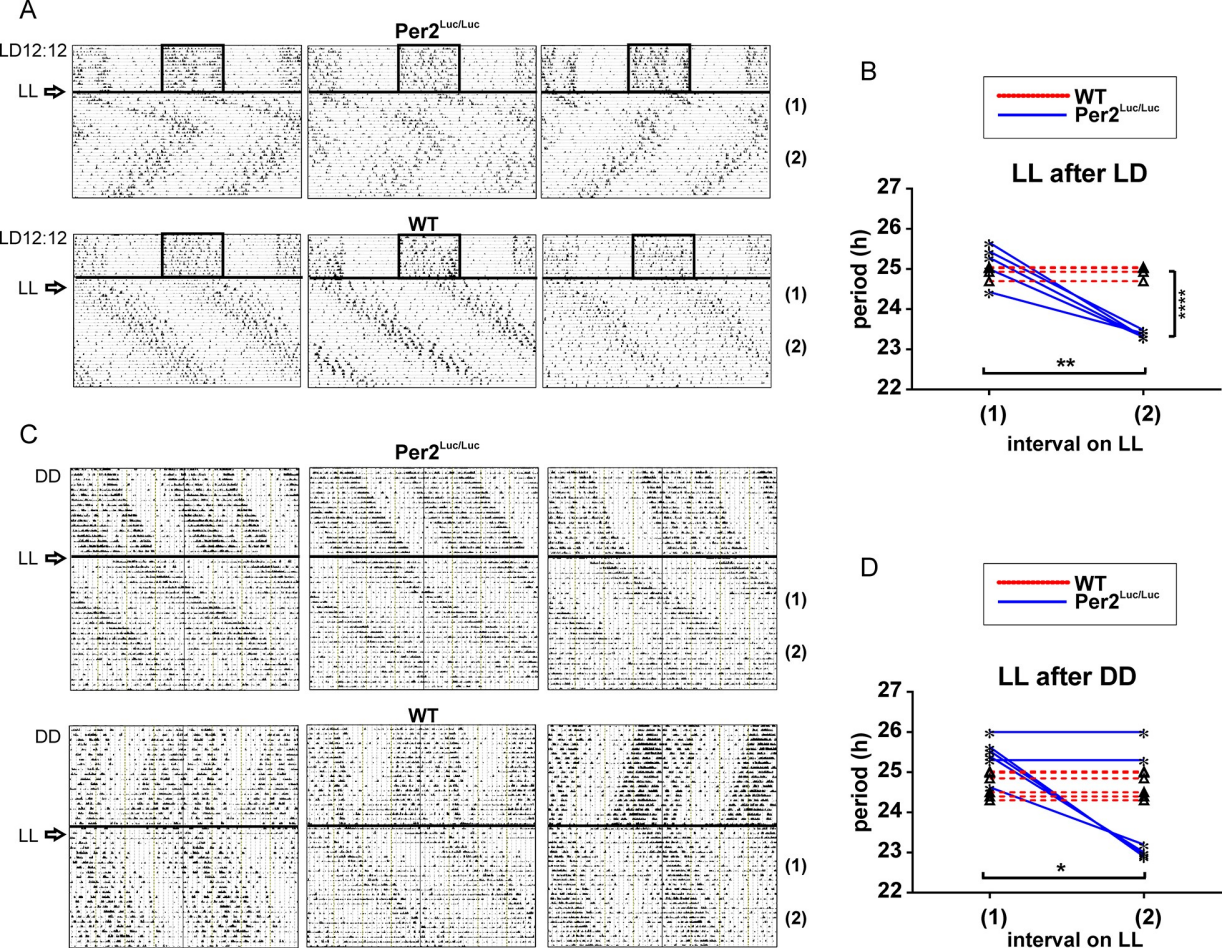
Genotype differences in OFA rhythms under LL following LD entrainment or DD exposure. **A**. Representative records of *mPer2*^*Luc/Luc*^ and WT control animals in LL following pre-exposure to LD12:12. **B**. Changes in periods of locomotor activity rhythm under LL exposure after entrainment to LD12:12. Periods were assessed in LL during the initial (1) and late (2) intervals (after 10 days in LL). *mPer2*^*Luc/Luc*^ mice show a long period (>24 h) for the initial interval (1) in LL, which spontaneously switches to a short period (<24 h) during the late recording interval (2), whereas WT mice exhibit the long period throughout the experiment. **C.** Representative records of WT C57Bl6 control animals and *mPer2*^*Luc/Luc*^ in LL following free-running in DD. **D.** Changes in periods of locomotor activity rhythm under LL after exposure to DD. Periods were assessed in LL during the initial (1) and late (2) intervals (after 2–3 weeks in LL). Periods in LL after pre-exposure to DD are long (>24 h) for both genotypes during the initial recording interval (1). During the late recording interval (2), the periods in some of *mPer2*^*Luc/Luc*^ mice spontaneously switch to a short period (<24 h) but retain long throughout the recording in all WT mice. * = *P* < .05; ** = *P* < .01; **** = *P* < .0001.

Representative OFA records from animals during the initial (1) and late (2) intervals following transition from LD12:12 directly to LL, are shown in **Fig 3A.** The comparison of mean period values (**Fig 3B**) revealed that the initial responses of both WT C57Bl6 (n = 6) and *mPer2*^*Luc/Luc*^ mice (n = 6) to LL were lengthening of average circadian periods (24.95 ± 0.15 and 25.16 ± 0.48, respectively). Thereafter, WT mice continued to express the >24 h period for the duration of this experiment while rhythms of the *mPer2*^*Luc/Luc*^ animals spontaneously switched to a period significantly shorter than the initial response (23.36 ± 0.08; *P* = .0012) after 2–3 weeks in LL (interval 2). As a result, the periods of the WT and *mPer2*^*Luc/Luc*^ animals became significantly different (*P* = .0001). Importantly, the short period in *mPer2*^*Luc/Luc*^ animals is maintained after several months in LL as reported previously [25].

When OFA was monitored in LL following exposure to DD, the latency for *mPer2*^*Luc/Luc*^ to express the short period rhythm of OFA was either prolonged relative to the animals released from LD, or the short period component was not detectable within the recording interval **(Fig 3C)**. Immediate responses of WT and *mPer2*^*Luc/Luc*^ mice to LL did not differ (WT: 24.68 ± 0.31, n = 6; *mPer2*^*Luc/Luc*^: 25.41 ± 0.46, n = 6) (**Fig 3C**; interval 1). Thereafter, WT animals expressed a >24 h period (24.68 ± 0.31) throughout the recordings (**Fig 3C and 3D** interval 2). In contrast some of the *mPer2*^*Luc/Luc*^ mice began to exhibit periods significantly shorter (23.89 ± 0.3; *P* = .0319), similar to animals transferred from LD, and some maintained the longer period (**Fig 3D**).

**Fig 4:**
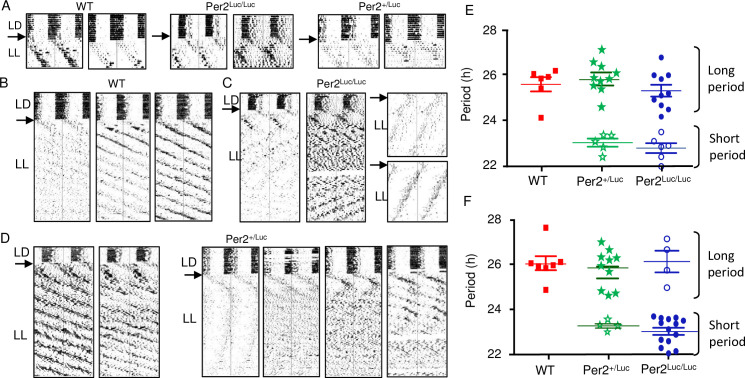
Period dissociation in LL detected by long tern recording of RWA. **A**. Initial responses of RWA to transition from LD12:12 cycle to LL. Two representative actograms from each genotype are shown. **B, C, D.** Examples of long term RWA in WT, *mPer2*^*Luc/Luc*^, and *Per2*^*+/Luc*^ mice, respectively. All animals were entrained to LD12:12 prior release into LL. Wild type animals **(B)** show the expected range of periods (all >24 h in LL); whereas homozygous *mPer2*^*Luc/Luc*^ mice **(C**) show dual rhythms (<24 h and >24 h in LL). (**D**) Heterozygous *mPer2*^*+/Luc*^ mice exhibit individual variance with patterns ranging between the wild type and mutant characteristics, as well as unpredictable arrhythmicity over time in LL. **E**. Period analysis of rhythm from the 3 genotypes in the first 7 days following transition from LD to LL. **Red, Green, Blue symbols** indicate significant periods of individual mice from each of the three genotypes. Closed symbols indicate the higher amplitude rhythm. Open symbols indicate a second significant rhythm with lower amplitude. No significant genotype differences were found among either long periods or short periods. **F**. Expression of short (<24 h) and long (>24 h) periods in LL determined 12 weeks following entry into LL (symbols shown as described in 4F). No significant differences between genotypes as in 4E. Long and short periods were significantly different within genotype for both *mPer2*^*Luc/Luc*^ and *Per2*^*+/Luc*^.

RWA recordings in LL presented similar differences between WT and *mPer2*^*Luc/Luc*^ to those seen in OFA recordings. Comparison of long-term RWA of all three genotypes upon release from LD12:12 to LL revealed that the initial response over the first 10 days, was the production of long period rhythms (>24 h) with no significant genotype differences observed **(**WT: 25.47 ± 0.34, *n* = 6; *mPer2*^*Luc/Luc*^: 24.77 ± 0.28, *n* = 10; mPer2^+/Luc^: 25.78 ± 0.23, *n* = 10) (for examples, see **Fig 4A**). In WT animals the >24 h periods were still evident after several months of recording in LL (**Fig 4B**). However, in the *mPer2*^*Luc/Luc*^ and *mPer2*^*+/Luc*^ strains, significant short period rhythms appeared in most records within the first 2 weeks in LL. Distinct long (>24 h) and short (<24h) periods were expressed either simultaneously or in alternating pattern over time. After a few weeks in LL, *mPer2*^*Luc/Luc*^ mice had predominantly <24 h periods **(Fig 4C)**. Heterozygote *mPer2*^*+/Luc*^ mice under identical conditions, showed a broad range of responses from individuals, exhibiting rhythm characteristics of either WT (**Fig 4D**, left panels) or *mPer2*^*Luc/Luc*^ (**Fig 4D**, right panels), or characteristics of both genotypes. Over the course of the recording, most heterozygous and homozygous *mPer2*^*Luc*^ animals switched between the expression of the longer and shorter phenotype, and many expressed both long and short components concurrently.

Because most long-term RWA records from *mPer2*^*Luc*^ mice showed evidence of developing dual periodicities, we quantified and compared both the long and short period rhythms of each genotype using chi-square periodogram analysis. As expected, the period difference between long and short components was highly significant for both *mPer2*^*+/Luc*^ (25.78 ± 0.23 h, *n* = 10 vs. 23.51 ± 0.20 h, *n* = 5, *P* = 1.9E-05) and *mPer2*^*Luc/Luc*^ (25.77 ± 0.28 h, *n* = 10 vs. 23.22 ± 0.13 h, *n* = 6; *P* = 0.0001). An analysis conducted for the early exposure to LL (over the 1^st^ 3–6 weeks), revealed no significant genotypes differences in either of the predominant periods (**Fig 4E**). Long periods were not different among the three genotypes, and short periods exhibited by some *mPer2*^*+/Luc*^ and *mPer2*^*Luc/Luc*^ mice were not different from each other. A second analysis conducted on activity after 2 months in LL did not show additional changes in periodicity except that the shorter component increased predominance in the *mPer2*^*Luc/Luc*^ mice (26.16 ± 0.55, *n* = 4 vs. 23.77 ± 0.28, n = 14; *P* = 4.7E-04) (**Fig 4F**), as was found in the OFA response (cf. **Fig 3B**).

### Circadian responses to light pulses are increased in *mPer2*^*Luc/Luc*^ mice

**Fig 5:**
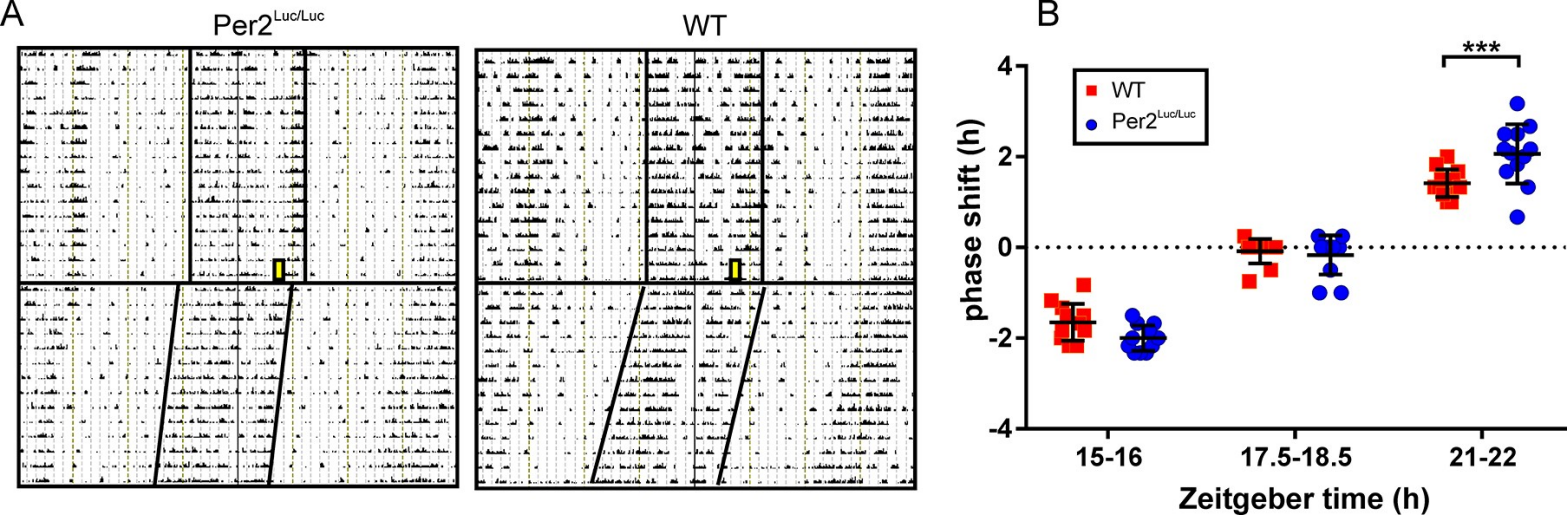
Phase dependent responses of OFA rhythms (phase shifts) to acute light exposure. WT and *mPer2*^*Luc/Luc*^ mice were entrained to LD12:12, exposed to a 1 h light pulse at different phases of the activity rhythms and released in DD. **A.** Representative actograms demonstrating the genotype-dependent difference in responses induced by light pulse administered at ZT21 (late night). **B.** Comparison of phase responses to light pulses between WT (n = 12) and *mPer2*^*Luc*^ (n = 12) mice in the early (ZT15), middle (ZT17.5) and late (ZT21) subjective night. Significant difference in phase responses between genotypes was found only at ZT21. *** = *p* < .001. Error bars = SEM.

Mice of both genotypes were maintained in LD12:12 then released into DD. Groups were exposed to a 1 h light pulse at one of the 3 different times during the final night, at ZT15 (early), ZT17.5 (middle), and ZT21 (late) night. Effects of the light pulses were assessed by phase shifts of the rhythms in the ensuing constant dark period. Representative actograms of WT and *mPer2*^*Luc/Luc*^ exposed to a light pulse at ZT21 are shown in **Fig 5A**. Analyses of behavioral responses in both genotypes (**Fig 5B**) revealed that a light pulse administered at ZT15 induced phase delays which were of about the same magnitudes (*P* = .0844) in WT (1.65 ± 0.41, n = 12) and *mPer2*^*Luc/Luc*^ (2.00 ± 0.28, n = 12), and a light pulse at ZT17.5 had no effect on the phases (WT: 0.08 ± 0.27, n = 12; *mPer2*^*Luc/Luc*^: 0.17 ± 0.43, n = 12; *P* = .6225). However, phase advances induced by a light pulse administered at ZT21 were significantly larger *P* < .001) in *mPer2*^*Luc/Luc*^ (2.06 ± 0.65, n = 12) compared to WT (1.42 ± 0.31, n = 12). The results suggest an increased responsiveness to light in *mPer2*^*Luc/Luc*^ mice, which was most prominent during the second part of the subjective night.

### *mPer2*^*Luc*^ alters behavioral activity profile and adaptation to scheduled food availability

**Fig 6:**
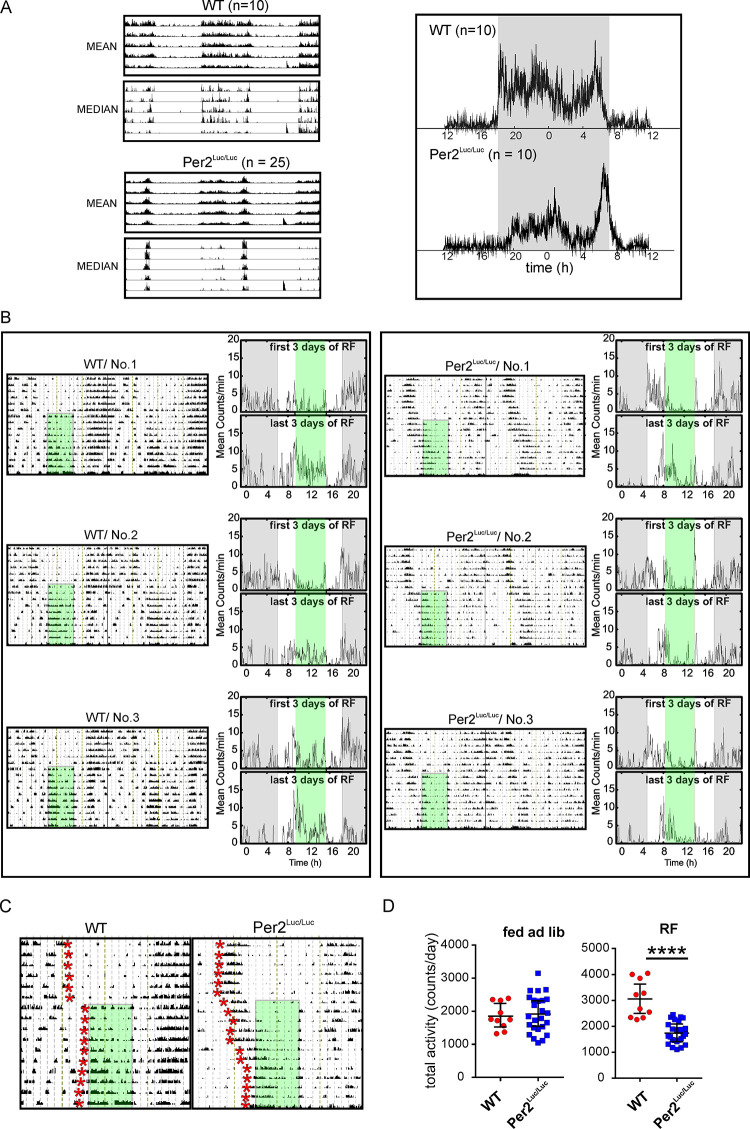
Entrainment of OFA with restricted food availability during the subjective day (RF). **A.** Activity profiles of mice entrained to LD12:12 cycles. Left panels: group-accumulated actograms of WT (n = 10) and *mPer2*^*Luc/Luc*^ (n = 25) mice calculated as activity average (upper pair) or median (lower pair). The short activity bout (red arrow) during the last inactive period (at ZT9) is the reaction to the first food removal at the beginning of the RF schedule. Right panels: group-composite daily activity profiles for 10 animals/genotype over the interval shown to the left. **B.** Representative double-plotted activity records from WT and *mPer2*^*Luc/Luc*^ animals in LD and exposed to RF for 10 days (green area). For each animal the raw activity recordings are shown in the actogram on the left, and the average activity profiles for the 1^st^ three days and last three days of RF are shown on the right. **C.** Representative single plotted actograms (from records in B, No.1 WT and No. 1 *mPer2*^*Luc/Luc*^) demonstrate genotype specific differences in the mode of behavioral adjustment to RF. The end of activity (WT) or beginning of the late-night activity bout (*mPer2*^*Luc/Luc*^) are depicted day-by-day as the red stars. In *mPer2*^*Luc/Luc*^ animals the late-night activity bout, but not the early activity bout, gradually entrains with the food timing. In WT mice the activity adjusts as published elsewhere.^67^ **D.** Total activity during *ad libitum* feeding did not differ between genotypes but was significantly higher in WT during RF. **** = *p* < .0001.

We examined whether LUC fusion with PER2 alters behavioral adaptation to misalignment of feeding and light/dark regimes (restricted feeding, RF). The WT (n = 10) and *mPer2*^*Luc/Luc*^ (n = 25) mice maintained under LD12:12 were first monitored for OFA under *ad libitum* feeding conditions. The behavioral rhythms of *mPer2*^*Luc/Luc*^ animals were more strongly bimodal than of WT mice with a distinct late night activity bout, as demonstrated by the group-cumulated mean and median activity records (**Fig 6A;** left side) and activity profiles (**Fig 6A**; right side) of WT and *mPer2*^*Luc/Luc*^ recorded just before the start of the RF regime. After exposure to RF with access to food for 6 h centered on the middle of the light interval (representative actograms are shown in **Fig 6B**), the adaptation of OFA proceeded differently in WT and *mPer2*^*Luc/Luc*^ mice. The WT mice terminated their nocturnal activity at the end of the dark phase (as under *ad libitum* conditions) and started to be active again with the food presence at ZT3-9. The activity declined after food removal (during ZT9-12) and the beginning of their nocturnal activity remained phase locked with the light/dark cycle. They developed food anticipatory activity and became active before food was provided at ZT3. In contrast, in *mPer2*^*Luc/Luc*^ mice the two activity bouts expressed under *ad libitum* conditions responded differently to RF. The late night activity bout gradually shifted into daytime and during the second part of the RF regime it became entrained with the food presence attaining a positive phase angle of entrainment (food anticipatory activity). The early night activity bout remained synchronized with the light/dark cycle in both genotypes (for detailed records shown in 6B, see **Fig 6C**). Although the total activity did not differ between both genotypes under *ad libitum* feeding conditions, increased activity in WT under RF conditions did not occur in it *mPer2*^*Luc/Luc*^ compared to WT mice leading to a significant genotype difference under RF (**Fig 6D**).

### Mathematical modeling recapitulates rhythmic behavioral changes in *mPer2*^*Luc/Luc*^ mice and identifies potential mechanisms

**Fig 7:**
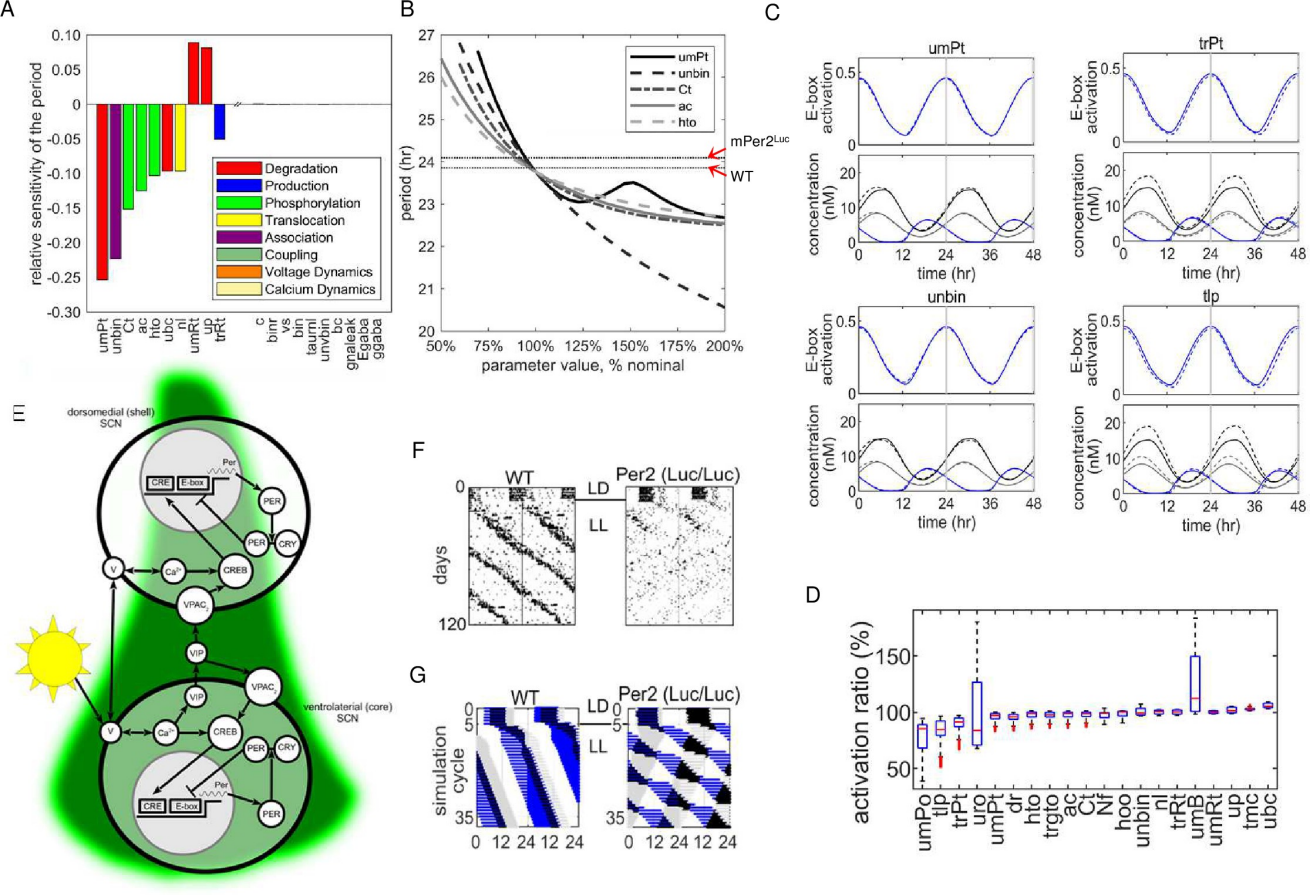
Extended mathematical model of SCN rhythm production. **A**. Parameter sensitivity analysis for single pacemaker cell period. Histograms represent the percent change for each parameter required to alter period in DD from WT to *mPer2*^*Luc/Luc*^. **B.** Sensitivity of period to the top 5 parameters. The model’s nominal base period is set at 23.8h for WT and 24.3 for *mPer2*^*Luc/Luc*^ as indicated by the two horizontal lines. **C.** Circadian patterns of dependent variables represented by changes in E-box occupation/activation and total concentrations of PER1, PER2, BMAL/CLOCK as a result of changing four parameters (*umPt*, *unbin*, *trPt*, *uro*) to obtain the mutant period. Solid lines = WT model. Dashed lines = *mPer*^*Luc*^ model. Time courses are normalized so that maximum E-box activation occurs at time = 0. Additional simulations are contained in **S2A–S2H Fig**. **D.** Comparison of E-box activation for simulations of WT and mPER2 Luc rhythms. The box and whisker plots show the distribution over one cycle: red lines are medians; blue boxes are first-to-third quartiles; black lines are extrema; and red dots are outliers. 100% = no difference between genotypes. **E.** Functional anatomy of the SCN pacemaker. The model is based on a primary transcription-translation feedback loop in all cells. The dynamics of the membrane potential V is coupled to the molecular clock through cytosolic calcium. The single cell model of the molecular clock is used for both the dorsal and ventral SCN regions. The regional difference is produced by the light input, and inter-regional communication is via VIP driving PER transcription through VPAC_2_ and the cAMP response element (CRE). VIP is produced and released from cells in the ventral SCN onto both shell and core cells. **F.** Representative actograms from WT and Per2^Luc^ mice housed in LL. **G.** Simulations using the two oscillator SCN model in LL with baseline parameters (left) and adjustment of *Per2* mRNA stability (*umPt*) to represent the presence of *Per2*^*Luc*^. **Parameter symbols**: *umPt*: degradation rate of *Per2* RNA; *unbin*: normalized unbinding rate for BMAL1-CLOCK/NPAS2 to *Per1/2/Cry1* E-box; *ac*: binding rate for PER1/2 to CK1ε/δ; *Ct*: total Ck1 concentration; *hto*: CK1ε/δ phosphorylation rate for PER2; *ubc*: degradation rate for BMAL1-CLOCK/NPAS2; *nl*: nuclear localization rate for proteins bound to PER; *trPt*: transcription rate for *Per2*; *mRt*: degradation rate for *Cry2*; *up*: degradation rate for CK1 phosphorylated PER; *vs*: production rate of cAMP when VPAC2R bound by VIP; *dg*: unbinding rate for PER2/REV-ERBs to GSK-3β; *cvbin*: binding rate of CRY1/2 to VPAC2R; *uro*: degradation rate constant for CRY1.

To address the potential mechanisms responsible for the behavioral changes in *mPer2*^*Luc*^ mice, we probed a mathematical model that describes cellular and biochemical processes to closely recapitulate the operation of the SCN clock [33–35]. The analysis was performed in two steps. First the sensitivity of the model to each of 111 parameters was tested to determine which could account for the genotype dependent period difference in DD. Then, simulations were run to determine how each of the variables (outputs) in the model responded to parameter changes that were sufficient to reproduce empirical behavioral data described above. Only a few of the parameters produced sufficient response of the model to account for the difference between WT and *mPer2*^*Luc*^ periods based on sensitivity alone (**Fig 7A**). Examples of individual period response curves in DD are shown in **Fig 7B** for five parameters to which the model is most sensitive to change. The average periods measured as RWA exhibited by WT and *mPer2*^*Luc*^ strains are indicated in the figure (arrows) for comparison. A complete list of parameters with the calculated period sensitivity of the expanded Kim-Forger-Stinchcombe model is provided in **S1 Table**.

From the model, circadian period in DD is most responsive, in descending sensitivity, to changes in (1) *Per2* transcript stability (*umPt*); (2) the separation (unbinding) of BMAL-CLOCK/NPAS2 from the PER1/2.Cry1 E-box (*unbin*); and (3) the binding of PER1/2 to CK1ε/δ (*ac*), and subsequent phosphorylation of PER2 (*hto)*. Periodicity was most sensitive to parameters that fell into three functional categories: Six parameters were associated with the regulation of PER2 abundance and activity, including factors that regulate active PER2 levels in the nucleus (*Per2* transcript longevity, *umPt*; PER2 phosphorylation/degradation, *Ct*, *ac*, *hto*, *up*; nuclear translocation, *nl*). Two others were associated with the stability of BMAL1-CLOCK/NPAS2 (*ubc*) or to the rate of its release from the PER1/2/CRY1 E-box (*unbin*). Two more were linked with transcription and degradation of *Cry2* (*trRt*, *umRt*). The model had a slightly lower sensitivity (ca. 1/5 maximum) to several parameters affecting PER2 interactions with CRY1/2 necessary for PER2 function in the TTFL. These included CRY1/2 translation rate (*tlr*), binding/unbinding rates of PER1/ with CRY1/2 (*ar*, *dr*), and CRY1/2 degradation (*uro*, *urt*).

Taken together the model was consistently highly sensitive to processes that affect PER2 negative regulation of E-box activation. Therefore, physiological changes linked directly to altered *Per2* transcription or protein regulation and/or interactions with CRY1/2 are sufficient to account for the long period seen in *mPer2*^*Luc/Luc*^ mice in DD. With the exception of *Cry2* regulation, circadian period is relatively insensitive to changes that would arise from second-order actions of PER2, such as changes in transcription rates of other E-box regulated genes. Notably, the period predicted by the model was not sensitive to the binding rate of CLOCK:BMAL1/NPAS2 to the PER1/2/Cry1 E-box (*bin*).

To examine the effects of altering individual parameter on the functionality of the DD model, we simulated the expression of rhythmic variables using each parameter adjusted to account for the different periods exhibited by WT and *mPer2*^*Luc/Luc*^ mice. Four representative simulations are shown in **Fig 7C** for rhythmic regulation of E-box state (occupied/not suppressed) along with total PER1, PER2, and BMAL1 concentrations in response to changes in *umPt*, *unbin*, *trPt*, and *uro*, all of which were identified previously in the sensitivity analysis and which are hypothetically affected directly by the genetic alteration. Comparative WT and *mPer2*^*Luc*^ simulations for 30 parameters simulated are shown in **S4–S11 Figs.** Parameters involved in PER2 production (*umPt*, *tlp*, *trPt*) exhibited high levels of E-box suppression (3 out of the top 7), along with significant increases in PER2 abundance (**Fig 7D**). The highest suppression of the E-box was produced by reducing *Per1* transcript degradation (*umPo*), which was associated with a reduction in PER2 level at all phases of the circadian cycle. Substantial increases in PER2 and nuclear fraction of PER2p:CRY1/2 were produced by reducing PER2 phosphorylation by CK1ε/δ (*ac*, *hto*), but with a small reduction of E-box activation. Finally, strong suppression of E-box activity was produced by changes in parameters that affect CRY1 and PER2:CRY1 stability. These include, a decrease in the degradation rate of CRY1 protein (*uro*) (**Fig 7, lower right**), and decreases in binding (*ar*) and unbinding (*dr*) of PER2:CRY1 along with reduction of PER2 and nuclear fraction of PER2p:CRY1/2.

To model the effects of LL, we started with the same parameters used for the DD simulations, and represented the SCN core and shell as two distinct pacemaker cell populations according to the current functional model of the SCN [36]. For LL simulation, the core pacemakers were sensitive to photic input represented by membrane potential and intracellular calcium and to intercellular coupling. The shell receives coupling signals from the core. Coupling signals are carried by VIP via the VPAC2 receptor in both core and shell.

The LL-induced dissociation of behavior into two rhythms, and the particularly short period exhibited in *mPer2*^*Luc*^ behavior, required further modification, where the SCN is represented by two populations of cells (**Fig 7E**). Under this arrangement, with only the core being directly responsive to photic signals, exposure to LL causes the rhythm in the core to move rapidly out of phase with the shell. For comparison samples of wheel running records from WT (left panel) and *mPer2*^*Luc/Luc*^ (right panel) responses to LL are shown in **Fig 7F**. In the WT model, exposure to LL produces overall period lengthening of overt behavior (**Fig 7G left panel**), with the output of the core (shown in blue) phase leading the shell (shown in grey). With the longer period produced by LL, entrainment of the shell pacemakers requires a larger phase angle between the two SCN regions.

The same parameter adjustment used to model *mPer2*^*Luc*^ period in DD (e.g. decreasing *umPt*), caused the core and shell rhythms to separate in the LL representation. In the LL model, the core generated a period >24 h, and the shell generated a component with a period <24 h, recapitulating the observed behavioral pattern in these mice (**Fig 7G right panel**). Whereas mutual entrainment between components fails in *mPer2*^*Luc*^ mice and in the model, each component exhibited relative coordination with the other, indicating bidirectional coupling. The short period from the shell suggests that nonphotic coupling signals from the core are producing both phase dependent advances and delays with advances being favored. Remarkably, relative coordination is more strongly indicated in the model by the long period rhythm under the influence of the core. This suggests that a robust rhythmic signal is produced in the shell that regulates rhythmicity in the core. In the model the shell → core coupling parameter represents average synaptic input to the core, the values being represented currently by inhibitory and excitatory GABA signaling, but can be adjusted readily to differentiate other potential signals such as arginine vasopressin [37, 38].

Two major processes that are implicated by the model as responsible for genotype differences in DD are the regulation of PER2 abundance and the ultimate influence of PER2 on CLOCK:BMAL1 E-box activation. To examine these potential causes we conducted two sets of experiments. In one we compared repression strengths of WT PER2 and PER2::LUC on CLOCK:BMAL1 transactivation of E-box containing promoters. In the second we examined effects of altering *Per2* stability.

### The *mPer2*^*Luc*^ allele modifies the PER2 repression of CLOCK:BMAL1

**Fig 8:**
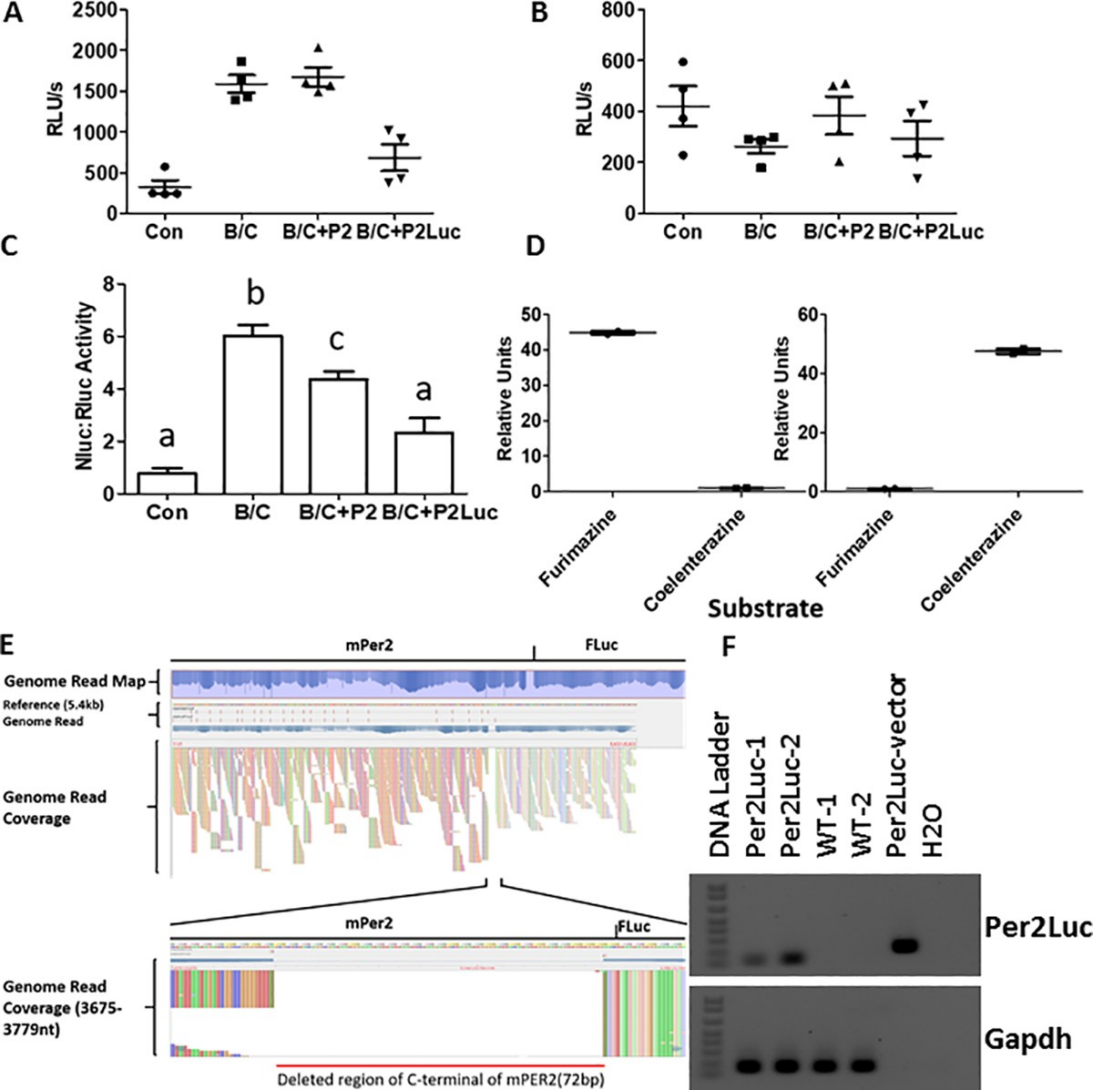
Mouse PER2 with C-terminal luciferase is a more potent transcriptional repressor of E-box *cis*-regulation in cell cultures. **A.** Activity of the NanoLuc luciferase reporter (P_E-box_NLuc) measured with the furimazine substrate in the co-transfected HEK293 cells. “Con” represents empty vectors (pCDNA3.1); B/C represents co-expression of BMAL1/CLOCK. Activity is expressed as RLU (Relative Light Units). Total amount of transfected plasmids was adjusted to the same concentration using empty vectors as needed **B.** Activity of the *Renilla* luciferase reporter (P_CMV_::RLuc) measured with the coelenterazine substrate in the same cell extracts as panel A. **C.** Activity of the NLuc luciferase reporter (P_E-box_) normalized by the *Renilla* luciferase control (P_CMV_). Statistical significance was analyzed by One-Way-ANOVA with Tukey’s post-hoc test at the p < 0.05 level as indicated by different letters (shared letters among groups represents no significant difference). Bars indicate Mean ±SEM; n = 4 for all groups. **D.** The furimazine and coelenterazine substrates do not cross react between the cells transfected with Nluc vs. Rluc. Left panel, cells that were transfected with only the P_E-box_NLuc plasmid were treated separately with each of the two substrates. Right panel, cells that were transfected with only the P_CMV_::RLuc plasmid were treated separately with each of the two substrates. The lower readings were normalized to 1 for comparison**. E**. From genome sequencing of *mPer2*^*Luc*^ mice, a C-terminal region of mouse Per2 is deleted in the *mPer2*^*Luc*^ mice. The unexpected 72 b.p. deletion at the C-terminal end of *Per2* is shown. **F.** Reverse transcription PCR detects a deletion of *Per2* mRNA in the *mPer2*^*Luc*^ mouse. The forward primer was designed to be in the m*Per2* gene upstream of the deleted region, and the reverse primer was designed to be the 5’ end of Firefly luciferase (Fluc). The “m*Per2*::*Luc* vector” that includes the entire m*Per2* gene fused to the *Fluc* gene without the 72 b.p. deletion was used as a positive control. Gapdh served as the control for cDNA integrity.

In a modified dual-luciferase/transient transfection assay using the luciferases NanoLuciferase (Nluc) and *Renilla* luciferase (Rluc), co-expression of CLOCK and BMAL1 increased the expression of an E-box reporter (P_E-box_NanoLuc) in transfected HEK293 cells when assayed for bioluminescence. CLOCK:BMAL1 activation of the E-box containing promoter was repressed more strongly by mPER2Fluc(P2Luc) than by mPER2(P2)(**Fig 8**). P_E-box_NanoLuc activity was assessed with the furimazine substrate (**Fig 8A**), and the *Renilla* luciferase reporter (P_CMV_::RLuc) is a control to assess transfection efficiency that is not affected by CLOCK:BMAL1, nor by co-transfection with either *mPer2* or *mPer2FLuc* in the same assay using the coelenterazine substrate (**Fig 8B**). When the activity of the P_E-box_ promoter (panel A) was normalized by the transfection efficiency (P_CMV_ reporter, panel B), the PER2::LUC fusion protein (P2LUC) clearly represses the P_E-box_ activity more strongly than does WT PER2 (P2) alone (**Fig 8C**). No cross reactions were found between the furimazine and coelenterazine substrates in the cells transfected with *NLuc* or *RLuc*, respectively (**Fig 8D**).

Whole-genome sequencing of DNA from mice verified that the targeted modification of *Per2* of our *mPer2*^*Luc/Luc*^ homozygous mouse corresponds to the Jackson Lab repository filing: “A luciferase gene and floxed neo^R^ cassette were inserted between exon 23 and the 3’ UTR,” (http://www.informatics.jax.org/allele/MGI:3040876). The insert was found as described, but unexpectedly, we discovered a 72 b.p. deletion at the 3’ end of the *Per2* coding sequence, resulting in the deletion of 24 amino acids from the C-terminus of PER2 **(Fig 8E and 8F)**. The same deletion was detected in independently established mouse colonies (Nashville, California, Cambridge). Overall, this confirmed the molecular identity and consistency of a mouse strain tested under experimental conditions. It also raised the question of whether the deletion at the C-terminus of PER2::LUC and the presence of the LUC protein interfere with CK1ε-mediated degradation of PER2 through action of the ubiquitin ligase adapter protein β-TrCP [39, 40].

### Modification of the 3’-UTR does not alter period

**Fig 9:**
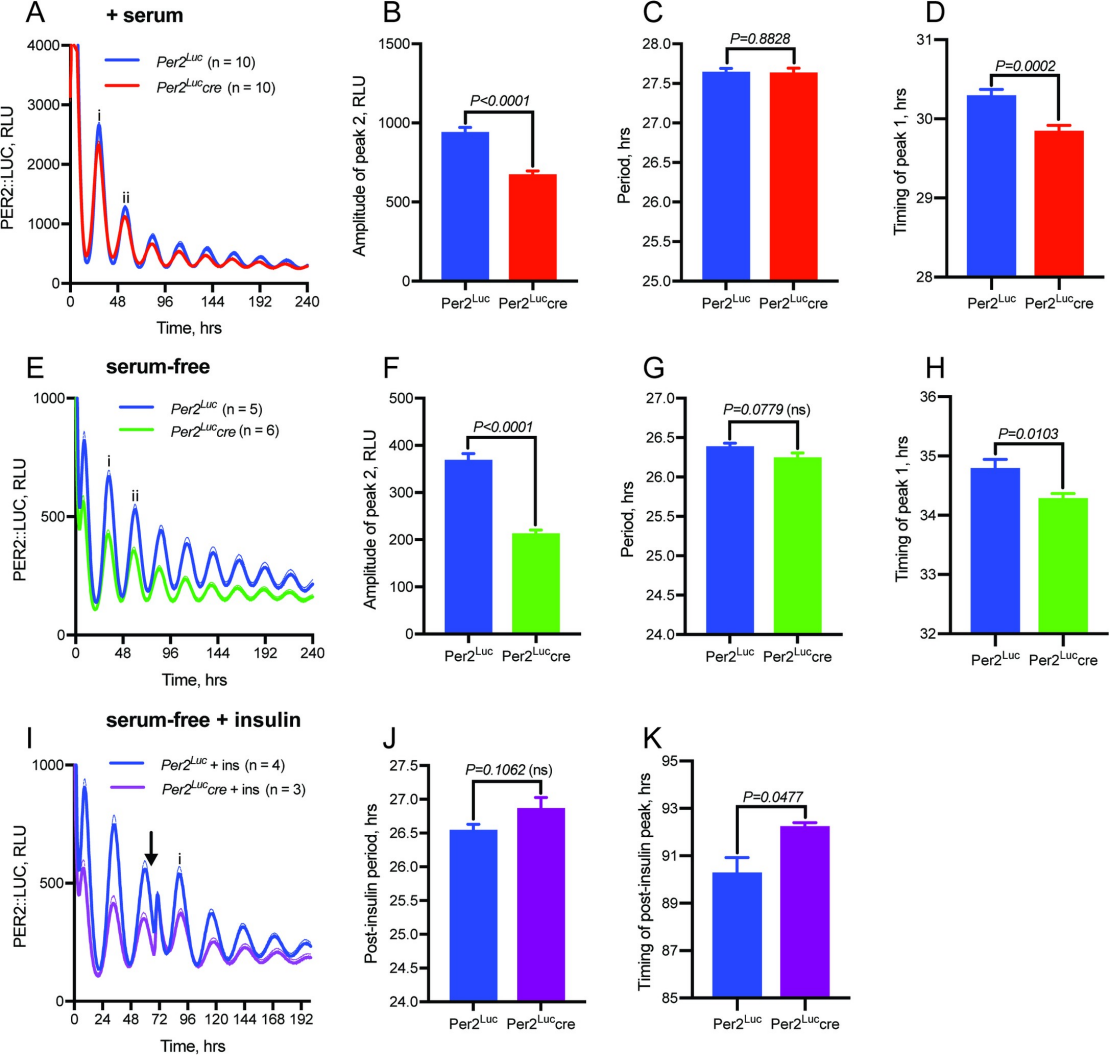
Presence of 3’-UTR neomycin resistance cassette modulates amplitude and phase-resetting in Per2^Luc^ cells. **A.** Bioluminescence recordings from *mPer2*^*Luc*^ and *mPer2*^*Luc*^*cre* lung fibroblasts in serum-containing media. **B.** Removal of the neomycin-resistance cassette from the *Per2* 3’-UTR using transiently-expressed Cre recombinase (*mPer2*^*Luc*^*cre*) reduces rhythm amplitude, as measured at peak ii, but **(C)** does not significantly influence period. **D.** Removal of the Neo cassette results in altered phase resetting in response to a media change at time 0, as measured by timing of the first circadian peak ‘i’. **E**. Representative bioluminescence recordings from *mPer2*^*Luc*^ and *mPer2*^*Luc*^*cre* fibroblasts in serum-free media shows comparable results to cells in serum-containing media, with **(F)** amplitude reduction at peak ii, **(G)** no change in period, and **(H)** an approximately 30 minute advance in timing of the first circadian peak in cells lacking the Neo cassette. **I.** Removal of the neomycin cassette still allows for insulin-induced PER2 expression, with **(J)** no significant difference in the subsequent period of the oscillation in serum-free media. **K**. Removal of the Neo cassette alters the phase of resetting following insulin, with an approximately 2 hour delay in the timing of the first circadian PER2::LUC peak following insulin. Arrow indicates addition of insulin.

As the 3’-UTR of mammalian transcripts is closely involved in the post-transcriptional regulation of gene expression, it is potentially an important regulatory element that has been altered in the *mPer2*^*Luc*^ mouse. Specifically, interactions of the 3’-UTR with regulatory proteins [41] and/or microRNAs [42] play important roles in transcript stability and amplitude, and in the translation of *mPer2* RNA to protein. We addressed the possibility that the observed behavioral changes might be due to an unspecified perturbation of the 3’-UTR function caused by the insertion of the neomycin resistance cassette (neo^R^), which is located between the end of the *Per2* coding sequence and the 3’UTR, and is flanked by loxP sites^1^. Here, we transiently transfected lung fibroblasts isolated from PER2::LUC mice with a CRE::GFP expressing plasmid, and then cell sorted for fluorescence. Genotyping showed very low presence of neo^R^ in those cells which had expressed CRE::GFP (*mPer2*^*Luc*^*cre*), suggesting that it had been successfully floxed out. Cells that had not expressed GFP during cell sorting did not show a clear reduction in neo^R^ levels. Recording of PER2::LUC bioluminescence from these cells (**Fig 9A**) revealed a clear reduction in amplitude in the cells which had expressed CRE (Per2^Luc^cre) and so lacked neo^R^ (**Fig 9B**) but no significant difference in the period of the oscillation (**Fig 9C**). The two groups did show a small but significant (~30 minute) difference in the phase to which they reset following a media change at time 0, as measured by the timing of the first circadian peak, marked as peak ‘i’ (**Fig 9C**). Cells recorded without serum showed lower overall levels of PER2::LUC bioluminescence than in serum-containing media (**Fig 9E**), but still exhibited a comparative amplitude reduction in those cells lacking neo^R^ (**Fig 9F**) with no significant difference in period (**Fig 9G**). Again, those cells lacking neo^R^ showed an approximately 30 minute advance in the timing of the first circadian peak (**Fig 9H**). Finally, cultures of *mPer2*^*Luc*^ and *mPer2*^*Luc*^*cre* fibroblasts were stimulated with 600nM insulin, which has been shown previously to affect phase, period, and rhythm amplitude in cultured cells through a mechanism dependent on miRNA regulation of the *Per2* 3’UTR [18]. Insulin transiently increased the LUC bioluminescence signal as well as the amplitude of the following peak of bioluminescence in all cultures, suggesting that the presence or absence of neo^R^ before the 3’UTR does not interfere with this acute miRNA regulation of *Per2* (**Fig 9I and 9J**). However, cells without neo^R^ displayed an ~2 h delay in the timing of the first circadian PER2::LUC peak ‘i’ following insulin addition (**Fig 9K**). Thus, excision of the neo^R^ cassette, whilst not influencing the free-running period of the PER2::LUC oscillation, reduced the overall amplitude of the rhythm and also subtly but significantly alters the nature of phase resetting following synchronizing cues.

## Discussion

In this study, we provide evidence that the *Luc* modification in *mPer2*^*Luc*^ mice results in broad changes in the regulation and overt expression of circadian rhythmicity. The results are significant because *Per2* plays several roles in the generation and regulation of rhythms in mammals and has numerous important functions beyond its role as a core clock gene [17]. Structurally the mPER2::LUC protein is mPER2 fused with a large LUC protein attached to its C-terminus, a region whose integrity is crucial for the stability of the protein and its interaction with CRY1/2 required for both CRY and PER longevity and nuclear translocation of PER2 [42, 43], and its central role in the TTFL of the molecular clock. A major finding of the study is that *mPer2*^*Luc*^ acts as a semi-dominant allele that is expressed in overt behavior as a circadian clock mutation. In support of this interpretation, we provide several lines of behavioral evidence that the reporter modification of the *Per2* gene affects its role in circadian timekeeping. Its impact on circadian behavior thus provides us with an insight into specific PER2 functions based on the finding that fundamental characteristics of circadian behavioral rhythms are altered in *mPer2*^*Luc*^ mice compared with WT mice: (1) In homozygous *mPer2*^*Luc/Luc*^ mice, free-running period is lengthened in DD and power of the rhythm estimation is higher. (2) In LL conditions, the behavior of *mPer2*^*Luc/Luc*^ mice dissociates into two components, one with a short period (<24 h) and the other with a long period (>24 h). (3) With variable latencies, the short period in *mPer2*^*Luc/Luc*^ mice in LL becomes the predominant circadian phenotype. (4) Heterozygous *mPer2*^*+/Luc*^ animals express unstable rhythms with a range of activity patterns resembling various intermediates between WT and *mPer2*^*Luc/Luc*^ phenotypes. (5) The temporal profile of behavior in *mPer2*^*Luc*^ mice exhibits strong bimodality. (6) *mPer2*^*Luc/Luc*^ behavioral responses to a light pulse during second half of the night are enhanced. (7) Daytime restricted food availability entrains the late nocturnal peak in the activity of *mPer2*^*Luc/Luc*^ mice.

Several neurochemical processes were identified using an adaptation of the Kim-Forger-Stinchcombe mechanistic model of the SCN that explain how an altered *Per2* gene could produce the observed behavioral period changes in DD. The use of the mechanistic model is a way to: (a) identify non-obvious mechanisms; (b) validate the hypothetical processes; (c) eliminate possibilities that don’t match published numerical data; and (d) identify the more likely of these possibilities. When examined for performance in DD, the model was sufficiently responsive to only 9 of the 111 parameters to account for the genotype difference in behavioral period given a 50–200% change in the individual parameter value. The majority of parameters used to model rhythm generation and regulation using this approach do not affect period unless a change in the parameter is far outside this range. Among these 9 parameters, 8 exert phase dependent influences over CLOCK:BMAL1/NPAS2 activity at the PER1/2/CRY1 E-box, through regulation of PER2 abundance and/or activity. This includes specifically reducing degradation rate of the *Per2* mRNA (*umPt*), lowering the rate of CLOCK:BMAL1/NPAS2 detaching from the E-box (*unbin*), and altering interactions of PER1/2 with regulatory proteins that influence PER1/2 stability (*Ct*, *ac*, *hto*, *up*) and nuclear localization (*nl*, *umRt*). The likelihood that *mPer2*^*Luc*^ could alter behavior via one or more of these mechanisms was substantiated with the finding that PER2::LUC is more effective than native PER2 as a suppressor of CLOCK:BMAL1-mediated E-box activation. The model is also sensitive to degradation of the CLOCK:BMAL1/NPAS2 structure (*ubc*); however, it is not apparent, (and we have not pursued), how modification of PER2 would alter this rate directly.

Importantly, high sensitivity of the model to variation in any particular parameter does not prove that the process is a causal factor. The relatively few parameters that significantly affect period is to be expected of a circadian regulatory system that must remain stable over time and resist transient changes in ambient conditions or even noise in the system. To pursue causal links between the *mPer2*^*Luc*^ interactions and the behavioral changes, we performed a semi-quantitative analysis of alterations in critical variables in the model that occurred when each parameter was adjusted to produce the *mPer2*^*Luc*^ period in DD. We judged each parameter on the requirements that the responsible processes must explain all of the empirical data (namely lengthen the period from *mPer2* to *mPer2*^*Luc*^ phenotype) as well as affect known regulation of and/or by the *Per2* gene–specifically *Per2* (*mPer2*^*Luc*^) RNA and protein levels, and E-box activation.

Several parameters to which the model is highly sensitive to change did not alter critical variables or were inconsistent with the current empirical data. These included *Cry2* mRNA degradation rate (*umRt*), *Cry2* translation rate (*trRt*), and nucleocytoplasmic (*nl*) shuttling. Parameters associated with CK1ε/δ phosphorylation of PER2 (*Ct*, *ac*, *hto*, *up*) had only small effects on E-box activation despite a predicted increase in levels of PER2pCRY1/2 in the nucleus in the late subjective day. In contrast five of the six parameters with the most robust and appropriate effect on the variables are associated with *Per2* transcription (e.g. ↑*trPt*) and translation (e.g. ↑*tlp*); however, there is no obvious feature of PER2::LUC that would explain an increase in the rate constants for these parameters. Interestingly the parameter with the greatest effect on E-box activity was the stability of the *Per1* transcript, but a decrease (↓*umPo*) required to lengthen period does not explain the direct effects introduced by *mPer2*^*Luc*^.

Nonetheless the effectiveness of several parameters in producing both period lengthening and suppression of E-box activation are consistent with PER2::LUC interference at the PER2 C-terminus. The participation of PER2 in the TTFL depends critically on an interaction with CRY1 in that the two molecules are mutually protected from ubiquitination and degradation, and PER2 is an effective repressor of E-box activation when bound to CRY1 [8, 44]. From the model, a decrease in CRY1 degradation (*uro*) predicts the greatest suppression of E-box activation of all the parameters with suppression peaking in the late subjective night and enhanced activity in the subjective day. The simulations also show that reduced CRY1 degradation is associated with lower levels of phosphorylated PER2 in the nucleus and cytoplasm. The significance of this result is supported by the finding that period also is sensitive to two parameters representing the binding and unbinding rates of PER2 to CRY1, *dr* & *ar*, respectively, which also produce appropriate alterations in key variables along with period lengthening, and could produce an apparent reduction in CRY1 degradation represented by *uro*. The altered behaviors of the variables, particularly E-box occupancy, suggest also that enhanced light induced phase shifts might be due to a relative increase in pCREB/CRE driven *Per2* activation in the context of lower background E-box activity in the late subjective night. The opposite may be the case in the subjective day when nonphotic phase shifts which are linked to reductions in pCREB mediated gene activity might be enhanced in the context of relatively higher background E-box activity [45, 46].

Therefore parsimonious explanations for the empirical data predicted from the model are that circadian period lengthening in DD is produced by (a) a relative increase in the abundance of *mPer2* gene products and/or (b) an increased stability of mPER2::LUC over mPER2 as partners in the TTFL. Higher mPER2::LUC levels over mPER2 could result from greater stability of the *mPer2*^*Luc*^ transcript over *mPer2* or increased translation. An increase in stability of mPER2::LUC brought about by extending the longevity of mPER2::LUC-CRY1 over PER2-CRY1 could occur via reduced phosphorylation/ubiquitination, protection of the proteins from degradation, and ultimately an increase in CLOCK-BMAL1 suppression^43^. This prediction of the model is supported by the increased PER2::LUC suppression of CLOCK:BMAL1 activity vs. PER2 in HEK cells. Potentially the presence of the LUC protein could interfere with CK1ε/δ activity in the C-terminal region and/or (2) promote binding of PER2 with CRY1. In both scenarios, this would increase the levels of PER2 (as PER2::LUC) available to suppress CLOCK:BMAL1 E-box activation [47, 48]. In addition the 72 bp deletion discovered in exon 23 of *Per2* truncates PER2 by 24 amino acid residues at the C-terminus. It is not clear whether this deletion is responsible for changes in PER2 activity; however, other targeted mutations in this region have been reported to increase PER2 stability [8]. Taken together, the body of evidence suggests that the *mPer2*^*Luc*^ modification has increased stability of the protein and produced a longer half-life over WT *Per2*.

A possible effect on period due to the retention of a floxed neo^R^ cassette in the *mPer2*^*Luc*^ reporter gene and an increased stability of the *mPer2*^*Luc*^ transcript [49, 50] was addressed. Removal of the floxed region in *Per2*^*Luc*^/cre cells significantly reduced the amplitude of *mPer2*^*Luc*^ rhythmicity, and phase shifts induced either by the media change at the start of bioluminescence recording or insulin administration during recording, but did not affect period. Therefore it seems unlikely that the longer period *mPer2*^*Luc*^ cells results from an increased transcript stability. However, cells *in vitro* as well as SCN explants are exposed to culture media that have no daily oscillation, whereas *in vivo* the same cells would be influenced by the circadian rhythmic environment of the living organism. Both insulin and IGF-1 receptors are expressed throughout the SCN [51, 52]. Moreover, rhythmic SCN explants respond to insulin with period changes when the stabilizing synaptic communication within the SCN [53] has been disrupted by pretreatment with tetrodotoxin [54]. Perhaps the small effect of the *mPer2*^*Luc*^ phenotype on circadian phase shifts in culture might produce significant period differences *in vivo*, where insulin and other potential nonphotic inputs exhibit circadian variation.

The LL condition presents an additional complexity to understanding the functioning of the clock in *mPer2*^*Luc*^ mice, namely because (a) constant illumination imposes a broad and continuous stimulation of the entire time-keeping system; and (b) responses to light are circadian phase dependent. During LL exposure, rhythmic behavioral components with two distinct periods emerged in *mPer2*^*Luc*^ mice and the relative dominance of either the long or short period changed during long-term recording. This pattern was detected earlier with OFA than RWA recording after release into LL. Neither the long nor the short period rhythms were genotype specific. The predominant *initial* response upon being placed in LL was a period >24 h in all animals (cf. **Figs 3 and 4**), as is commonly seen in mice [55, 56]. The major genotype differences became apparent when free-running rhythms with short periods emerged in both *mPer2*^*+/Luc*^ and *mPer2*^*Luc/Luc*^ animals but not in WT. The *mPer2*^*Luc*^ allele dose did not alter either long or short periodicity *per se* in LL but did affect the predominance of short period expression in RWA. Individual heterozygous animals expressed circadian rhythmicity ranging from a WT to a homozygous, *mPer2*^*Luc/Luc*^ phenotype in LL, similar to the range of phenotypes found in DD. A question that remains is whether the unstable behavioral phenotype of heterozygous mice arises from variable (and unstable) expression of *Per2* and *mPer2*^*Luc*^ among pacemakers or unstable coupling between core and shell oscillators.

We questioned whether the dissociation of two rhythmic components in LL reflects a type of “splitting” phenomenon seen in other rodent models. Evidence for dual oscillator organization in circadian systems is abundant in the mammalian literature [57]. A splitting phenomenon formed the basis of a model where the temporal organization of activity was produced by two mutually coupled oscillators, one which tracked dawn (a morning oscillator, M) and the other which tracked dusk (an evening oscillator, E), which could become desynchronized temporarily, then resynchronize in antiphase by exposure to LL [58]. Phenomenological mathematical models have successfully described this behavioral splitting of Syrian hamster rhythms in LL [59, 60]. The pacemaker responsibility has been attributed to outputs of the two bilateral SCN [61–63]. It also has been reported that Per1:Gfp transgenic mice housed in LL, produce either a long period; splitting of 24 h rhythms into two bouts or arrhythmicity [55]. In this case arrhythmicity was attributed to desynchrony among pacemaker cells within the SCN.

Behavioral records from our *mPer2*^*Luc*^ mice show clearly two distinct behavioral rhythms with different periods. The first activity onsets in LL were strongly delayed, and the subsequent long period free-run emerged from a point at the end of the final subjective night in prior DD or LD. Although the short period rhythms were not immediately obvious upon entering LL, the free-run, when projected back to the prior LD records, also started from the latter half of the subjective night. Similar patterns were produced in OFA and RWA. There is no evidence that the right and left SCN are functionally distinct prior to LL, therefore because the light input signal is restricted to the SCN core, a likely mechanism responsible for LL-induced rhythm dissociation in *mPer2*^*Luc*^ mice is the anatomical and functional division of the SCN into shell and core regions [64]. The clock in the core region can be entrained directly by light so that the two regions can be driven out of phase by LD cycles when the Zeitgeber period is beyond the limits of stable entrainment [65]. In rats the SCN responds to acute exposure to continuous light with a rapid delay of cFOS and PER1 rhythmic expression in the core (ventrolateral SCN) but not in the shell (dorsomedial SCN) [66]. This is consistent with the behavioral responses of *mPer2*^*Luc*^ mice being placed in LL which is predicted to drive the oscillation in the core separately from the shell, resulting in delay in activity onset followed by its separation into two rhythmic outputs from the SCN. The same hypothesis has been proposed to explain a splitting phenomenon exhibited by CS mice held in DD [36].

Using the modified Kim-Forger-Stinchcombe mathematical model [33–35], we viewed the SCN based on two sets of coupled oscillators with one representing the SCN shell, and the other the retinorecipient core. In our model, coupling between the two oscillator ensembles was a bidirectional synaptic relationship representing an average inhibition and excitation. VIP communication was unidirectional from core to shell with both core and shell cells receiving VIP input. Using the parameter values derived for rhythm production in DD, under the consideration of SCN physiology and anatomy, the mathematical model successfully reproduces the observed behaviors of *mPer2*^*Luc*^ mice in LL. As a result of the lengthened circadian period of *mPer2*^*Luc*^ mice in DD, the oscillator in the core (which phase leads the shell in WT in an LD cycle), assumes a delayed phase angle of entrainment relative to the shell. This produces a greater coherence of core and shell oscillators represented by the simulations shown in **Fig 7F**. When this is combined with LL input to the SCN core, the outputs of the two regions have sufficiently different periods that stable entrainment cannot occur. The dissociation results in the expression of two circadian rhythms that are mutually coupled as is apparent from the period fluctuations (relative coordination) exhibited by both rhythms. Interestingly, the fluctuations suggest that the core is more responsive to the input from the shell than vice versa.

The core-shell model also may offer insight into the temporal structure of daily behavioral rhythms. Period lengthening in DD, introduced by PER2::LUC, produces greater congruence of core and shell outputs. Theoretically this could exaggerate bimodality seen in *mPer2*^*Luc*^ mice by more strongly defining the divisions between subjective night and day. The larger phase angle in WT means the start of the subjective day produced by core cells would occur ahead of that of the shell, potentially suppressing overt behavior in the late night that would be permitted by the congruence of the shell and core. The phase advance of the shell oscillation is consistent with increased response to light in the late subjective night during photic entrainment.

The *mPer2*^*Luc*^ response to an RF schedule also is consistent with a dual oscillator model. However, food anticipation can be produced in arrhythmic rodents with SCN lesions, indicating the presence of one or more distinct food entrainable oscillators [67]. The SCN is less sensitive to shifts in feeding schedule than peripheral oscillators [68] indicating that the robustness against feed-fast cycles is likely due to strong independence and interneuronal coupling [48], which is diminished in LL [55]. Indeed, under LL conditions the SCN may become partially sensitive to RF, suggesting a potential resynchronization of cellular clock via nonphotic input [69]. Nonetheless, it seems most likely that extra-SCN regions where the PER2 function is also altered [17] may participate in modulation of behavioral responses to RF. As discussed above, this could be explained by altered responsiveness due to an increase in background E-box activation in the subjective day. Therefore, we cannot exclude the possibility that compared with WT, the increased bimodality driven by the SCN of *mPer2*^*Luc*^ mice enhances an underlying behavioral response to RF. In this context, it will be important to examine the responses to RF using both OFA and RWA as wheel running has a nonphotic feedback influence on circadian period [21] and profile of nocturnal goal directed behavior [70].

Several examples are presented in these data which allude to differences in bimodal expression of behavior among the *mPer2* genotypes examined. These include genotypic differences in the temporal profiles of locomotor activity (cf. **Figs 1 and 2**), dissociation of two components in LL (cf. **Fig 4**), entrainment profiles to RF schedules (cf. **Fig 6**, and the division of the SCN into light-sensitive and -insensitive components (cf. **Fig 7**). It seems reasonable to assume bimodality based on activity data that produces two or more peaks, and there is substantial support in the published literature that the mammalian circadian system is anatomically and functionally bimodal [58–66]. However, although behavioral patterns may be modulated by underlying circadian influences, the raw data–the quantifiable locomotor activity from individuals–also is affected by incidental factors such as age, health, motivation, attention, prior conditions, sensory inputs, and variance among the instruments (especially running wheels) used to gather data. Therefore, while differences in behavioral patterns which have arisen with the LUC modification PER2 suggest changes in underlying bimodal mechanisms, the interpretation from quantitative analysis should be approached with caution. Nonetheless, it is tempting to speculate that several activities of *Per2* in addition to its role in circadian rhythm generation, are altered in *mPer2*^*Luc*^ mice.

## Conclusions

The presence of the *mPer2*^*Luc*^ allele produces widespread changes in circadian rhythm regulation which include changes in free-running period in DD and LL, reduced stability of circadian period, and altered responses to photic input and RF schedules. Based on qualitative and quantitative analysis of the mathematical model, expanded to differentiate light responsive and nonresponsive regions of the SCN, the behavioral changes and other empirical data can be explained by the presence of PER2::LUC acting to increase the stability of the PER2:CRY1 dimer which results in increased negative feedback on CLOCK:BMAL1 E-box activation as part of the circadian clock TTFL.

The altered patterns of behavioral responses in DD and LL may be explained by the known regional differences in circadian functionality within the SCN, suggesting that caution should be exercised when interpreting tissue and behavioral level rhythmicity as a single, uniform output from this nucleus. The instability of period in DD found in heterozygous mice further emphasizes the need for additional caution when interpretation of experimental data relies on circadian responses to stimulation. Neither mathematical modelling nor biological experimentation have been engaged as yet in the pursuit of this explanation; however, several interesting possibilities exist with the most prominent being that mPER2 and mPER2::LUC produce differential suppression of the two *Per2* alleles. Finally, a genotype difference was found in the initial period expressed when released into DD, that is, *mPer2*^*Luc*^ mice has a shorter initial period than WT. Though this difference is small, the change in response is consistent with a genotype differentiated decompression of the rhythm as an aftereffect of prior entrainment. Although results of this study demonstrate that clock mutation *mPer2*^*Luc*^ could confound some experimental results due to the reporter’s effect on rhythm generation, this model still provides a valuable tool for explorations into the functional organization of mammalian clocks.

## Methods and materials

### Ethics statement

All procedures were approved by either (a) the Animal Care and Use Committee of the Institute of Physiology of the Czech Academy of Sciences, in agreement with the Animal Protection Law of the Czech Republic, as well as the European Community Council directives 86/609/EEC and 2010/63/EU, or (b) the University of Toronto Animal Care Committee according to the guidelines of the Province of Ontario and the Canadian Council on Animal Care.

### Animals

Adult C57Bl6 (wild type, WT) mice and reporter homozygote *mPer2*^*Luc/Luc*^ and heterozygote *mPer2*^*+/Luc*^ mice on a C57Bl6 background, were used in the study. Experiments were conducted at two facilities to identify and account for potential nonspecific procedural differences that might affect interpretation of the results. For comparison, inbred animals of each strain were obtained from two different breeding colonies, one located in Czech Republic (Institute of Physiology of the Czech Academy of Science, Prague) and the other in Canada (Biological Sciences Facility (BSF), University of Toronto). To avoid period variance due to estrous cycles, only male animals were used in these studies. The original breeding stocks of both cohorts were derived from strain B6.129S6-Per2tm1Jt/J, IMSR_JAX:006852, (Jackson Laboratories Bar Harbor, USA). Additionally, the WT C57Bl6 and homozygote *mPer2*^*Luc/Luc*^ mice were crossed to obtain heterozygote *mPer2*^*+/Luc*^ mice (produced at the BSF in Toronto). During experiments in both facilities, animals were housed individually in temperature-controlled (21 ± 2°C) animal rooms and had free access to food and water throughout each experiment. Light/dark cycles when applied, were 12 h of light and 12 h of darkness (LD12:12). Lighting schedules were determined according to local time at each facility with lights on defined as Zeitgeber time 0 (ZT0) and lights off as ZT12. For LD and LL experiments light intensities were between 90 and 120 lux measured at each cage floor. For cre knockout experiments, *mPer2*^*Luc/Luc*^ mice were bred in a specified pathogen free barrier facility (MRC LMB, UK) from a stock originally supplied by Joseph Takahashi (University of Texas Southwestern).

### Locomotor activity monitoring

Two methods were used to analyze the effect of PER2::LUC on circadian locomotor behavior. In the Prague facility, behavior was recorded as spontaneous OFA using infrared activity detectors, and in the Toronto facility as goal directed behavior using RWA. For detection of OFA, mice were maintained in cages equipped with infrared movement detectors attached above the centre of the top of the cage. A circadian activity monitoring system (provided by H.M. Cooper, INSERM, France) was used to accumulate activity into one minute bins. The parameters of the activity rhythms were analysed using the ClockLab toolbox (Actimetrics, Wilmette, IL, USA). The RWA was monitored using rotations of a running wheel in each cage. These were accumulated into six minutes intervals using VitalView (Phillips-Respironics, Bend, Oregon). Periodogram analyses of these data were performed using Actiview (Phillips-Respironics, Bend, Oregon). Double-plotted actograms and chi-squared periodograms were generated using ClockLab to evaluate activity levels, calculate period and amplitude (power of the period estimation) of the rhythms. Cage sizes: OFA: 43L x 27W x 23H cm; RWA: 30.3L x 20.6W x 26H cm.

Analyses of free-running periods were performed using animals that were synchronized to the LD12:12 cycle and then released into constant conditions according to the following protocols: For DD, the darkness was extended following the dark interval in the LD12:12 cycle (LD → DD). For LL, the light period of the LD12:12 cycle was extended (LD → LL). For the animals that were switched to LL after free-running in DD (LD → DD → LL), the transition to LL occurred during the early inactive phase within a window of 3 h, due to individual differences in the DD periods.

### Circadian phase dependent responses to light

Light-induced phase shifts were assessed as *Aschoff Type II* method [71]. WT and *mPer2*^*Luc/Luc*^ mice entrained initially to a LD12:12 lighting schedule were monitored for OFA as described above. One-hour pulses of light were delivered to 12 mice of each genotype by switching on the room lights at one of three time points (ZT15, ZT17.5 or ZT21) during the final dark phase in LD. Thereafter, the mice remained in DD for 3 weeks. The resulting phase shift was calculated by comparison between the eye fits of OFA onsets at least 10 days before and 12–14 days after the light pulse. To avoid any bias, the phase was determined by two persons blind to the procedure.

### Restricted feeding (RF) schedules and food anticipatory activity (FAA)

Mice of both genotypes (WT, n = 10; *mPer2*^*Luc/Luc*^, n = 25) maintained in LD12:12 were used for testing their responses to a restricted feeding (RF) schedule. In the RF protocol, access to food was limited to a 6 h-interval in the middle of the light phase of the LD12:12 cycle (ZT3 –ZT9). The RF schedule started by removal of the food at ZT9 and was maintained for 10 days. OFA under the RF schedule was analyzed for the quantity and temporal distribution of activity using ClockLab.

### *mPer2*^*Luc*^ sequencing

Genomic DNA from homozygous *mPer2*^*Luc/Luc*^ mouse was extracted and purified (A1120, Promega). The genomic DNA was measured for quantity using Qubit fluorometry (Invitrogen) and integrity by running on 2% agarose gel. After quality control, genomic DNA from the homozygous *mPer2*^*Luc/Luc*^ mouse was sequenced on the Illumina HiSEQ 3000 for a targeted 50X coverage of the entire genome. Sequencing was performed by the Vanderbilt Technologies for Advanced Genomics. Raw sequence data were processed and then aligned with published sequences of mouse *Per2* (NCBI CCDS35663.1) and firefly *luciferase* (*Luc*+, pGL3, Promega), using the Tablet program (Aberdeen, UK).

### Transient transfection assay of PER2::LUC vs. PER2 activity

HEK293 cells were co-transfected with a combination of five plasmids driving expression of Per2 or Per2-FLuc, NanoLuc (PE-box-NLuc), Renilla Luciferase (PCMV-RLuc), Bmal1, and Clock (Bmal1 and Clock have no luciferase tags). PCMV is the standard CMV promoter used in the Dual-Luciferase assay, and PE-box is a synthetic promoter that contains 3 canonical E-box motifs (CACGTG) in tandem. We modified the standard transient transfection Dual-Luciferase assay [72] to use NanoLuc instead of FLuc because the presence of FLuc on PER2::FLuc interferes with the standard Dual-Luciferase assay. Coelenterazine substrate was used to visualize RLuc with the Dual-luciferase kit (E1910). Furimazine substrate was used for NLuc with the Nano-Glo kit (N-1610). Transfected cells were extracted with 1X PLB lysis buffer (Promega E-194A) one day (24 h) after transfection, and measurements for the various luciferases and assessment of potential cross-reactivity were conducted using the same amount of cell extract. The forward primer for *mPer2Luc* (<CATCGATGTGACAGGCTGTG>) was designed to be upstream of the deleted region. The reverse primer (<GCCGGGCCTTTCTTTATGTT>) was designed to be at the 5-prime end of firefly luciferase (FLuc).

### *Per2*^*Luc*^ cre methods

*mPer2*^*Luc*^ mice were originally supplied by Joseph Takahashi (University of Texas Southwestern) and subsequently bred locally in a specified pathogen free barrier facility. Mouse fibroblasts were obtained from lung tissue of adult *mPer2*^*Luc*^ and immortalised by serial passage [73]. Removal of the neomycin cassette from the genomic Per2^Luc^ locus was achieved by transient expression of cre-recombinase. Briefly, cells were trypsinised and resuspended in buffer containing a CRE::GFP plasmid (kind gift of Ernesto Ciabatti) and electroporated using a Neon transfection system (ThermoFisher) as per manufacturer’s instructions. Cells were allowed to grow in a single well of a 6-well plate for 72 h. They were then trypsinised, resuspended in serum-free media and cell sorted to identify cells expressing CRE::GFP. Both positive and negative populations were reseeded in standard culture medium (high-glucose (27.8mM), glutamax-containing DMEM (GIBCO) supplemented with 10% serum (HyClone FetalClone III, Themofisher) and pen/strep), expanded and passaged as normal. Removal of the neomycin cassette was confirmed by genomic PCR.

For experimentation, cells were grown to confluence (passage no. <40) in 35mm experimental dishes in standard culture medium. Confluent cultures were kept for up to 4 weeks with media refreshed every 5–7 days. Before the start of recording, cells were synchronised by external temperature cycles of 12h 32°C followed by 12h 37°C for a minimum of 72h, then changed to “Air Media” (Bicarbonate-free, DMEM, 5mg/mL glucose, 0.35mg/mL sodium bicarbonate, 0.01M HEPES, 2 μg/mL pen/strep, 1% Glutamax, 1mM luciferin, pH 7.4, 350 mOsm); adapted from Hastings et al. [74] with or without serum and NS21 [49] and dishes sealed. Cells were then transferred to a Lumicycle luminometer where bioluminescent activity was recorded at 10 minute intervals. Insulin was dissolved in dilute HCl to form a 10mg/mL stock solution and stored at -20°C in aliquots. Before use, this was further diluted to 1mg/mL in cell culture media and added to cells under isothermal conditions to a final concentration of 600nM.

### Mathematical modeling

An integrated mechanistic model was created by combining the detailed molecular clock model of Kim & Forger^33^ describing transcription, translation, translocation, degradation, and phosphorylation of the clock components; the electrophysiology model of Diekman et al. [75] describing sodium, potassium, and calcium currents, with average SCN synaptic connectivity inferred from Stinchcombe et al.^34^; and the intercellular chemical coupling from DeWoskin et al. [34]. Parameters were chosen from published data so that long term oscillations with periods representing the C57Bl6 background mouse strain, were produced when light response and cell-cell coupling parameters were set to zero (i.e. representing average free-running period of single cells in DD). Sensitivity of the period to change in individual parameters was determined by computing the period in many simulations with each parameter varied between 50% and 200% of its initial (baseline) value.

Coupling among pacemaker cells was represented by rhythmic release of vasoactive intestinal polypeptide (VIP) and response of the VPAC2 receptor. Differences in rhythm production in the SCN core (ventrolateral SCN in rats; vlSCN) and shell (dorsomedial SCN in rats; dmSCN), are represented by two groups of essentially identical cells. One group, representing the retinorecipient cells of the SCN core, is influenced by light input [40] represented in the model by glutamate input mediated by the Ca^2+^/CREB signaling pathway.

The model was simulated using custom written code in MATLAB 9.6.0 (The Mathworks, Inc. Natick, MA) and C11. The numerical methods are described in DeWoskin et al. (2014) [34]. Simulation code, representing a complete description of the model, is available upon request.

### Statistical methods

For analyses of behavioral activity, free-running period for individual subjects was determined using chi-squared periodogram analysis (*ClockLab* software, Actimetrics, Wilmette, IL) for relevant sections of an actogram. Statistical comparisons were performed using *Prism* software (GraphPad Sofware, San Diego, CA). Significant group differences were identified with 2-way ANOVA (genotype x lighting treatment) and Tukey’s post hoc method or by unpaired t-test (genotype) with Welch’s correction. Within subject correlation analysis of period stability over time was performed by calculating Pearson’s R for paired data, with significance correlations identified with a Mann-Whitney U Test. Data are expressed as mean ± SEM and significance was assumed at *P* < 0.05.

## Supporting information

S1 Fig Open field activity of WT mice in LD and DD. Locomotor activity of wild-type (WT) mice (n = 11) recorded as spontaneous open field activity using infrared activity detectors. Mice were entrained to light/dark cycle LD12:12 (LD) and then released into constant darkness (DD) (arrow).
(PDF)

S2 Fig Open field activity of *mPer2^Luc/Luc^* mice in LD and DD. Locomotor activity of *mPer2*^*Luc/Luc*^ mice (n = 16) recorded as spontaneous open field activity using infrared activity detectors. Mice were entrained to light/dark cycle LD12:12 (LD) and then released into constant darkness (DD) (arrow).
(PDF)

S3 Fig Open field activity of WT and *mPer2^Luc/Luc^* mice in LD and DD and LL. Locomotor activity of wild-type (WT) mice (n = 6) and *mPer2*^*Luc/Luc*^ (n = 6) recorded as spontaneous open field activity using infrared activity detectors. Mice were entrained to light/dark cycle LD12:12 (LD), and then released into constant darkness (DD) followed by constant light (LL).
(PDF)

S4 Fig Responses of critical variables during simulated WT and *mPer2^Luc^* rhythm production. WT period = 23.8 h; mPer2^Luc^ period = 24.1 h. Time dependence of model output variables. The effects on E-box occupation/activation and PER1, PER2, BMAL/CLOCK total concentration as a result of changing each parameter to obtain the altered period in DD. Solid lines = WT model. Dashed lines = mPer2Lucmodel. Time courses are normalized so that maximum E-box activation occurs at time = 0. Common Y-axis labels are indicated on the left panels. See S1 Table for parameter definitions.
(PDF)

S5 Fig Responses of critical variables during simulated WT and *mPer2^Luc^* rhythm production. WT period = 23.8 h; mPer2^Luc^ period = 24.1 h. Time dependence of model output variables. The effects on E-box occupation/activation and PER1, PER2, BMAL/CLOCK total concentration as a result of changing each parameter to obtain the altered period in DD. Solid lines = WT model. Dashed lines = mPer2Lucmodel. Time courses are normalized so that maximum E-box activation occurs at time = 0. Common Y-axis labels are indicated on the left panels. See S1 Table for parameter definitions.
(PDF)

S6 Fig Responses of critical variables during simulated WT and *mPer2^Luc^* rhythm production. WT period = 23.8 h; *mPer2^Luc^* period = 24.1 h. Time dependence of model output variables. The effects on E-box occupation/activation and PER1, PER2, BMAL/CLOCK total concentration as a result of changing each parameter to obtain the altered period in DD. Solid lines = WT model. Dashed lines = mPer2Lucmodel. Time courses are normalized so that maximum E-box activation occurs at time = 0. Common Y-axis labels are indicated on the left panels. See S1 Table for parameter definitions.
(PDF)

S7 Fig Responses of critical variables during simulated WT and *mPer2^Luc^* rhythm production. WT period = 23.8 h; *mPer2^Luc^* period = 24.1 h. Time dependence of model output variables. The effects on E-box occupation/activation and PER1, PER2, BMAL/CLOCK total concentration as a result of changing each parameter to obtain the altered period in DD. Solid lines = WT model. Dashed lines = mPer2Lucmodel. Time courses are normalized so that maximum E-box activation occurs at time = 0. Common Y-axis labels are indicated on the left panels. See S1 Table for parameter definitions.
(PDF)

S8 Fig Responses of critical variables during simulated WT and *mPer2^Luc^* rhythm production. WT period = 23.8 h; *mPer2^Luc^* period = 24.1 h. Time dependence of model output variables. The effects on E-box occupation/activation and PER1, PER2, BMAL/CLOCK total concentration as a result of changing each parameter to obtain the altered period in DD. Solid lines = WT model. Dashed lines = mPer2Lucmodel. Time courses are normalized so that maximum E-box activation occurs at time = 0. Common Y-axis labels are indicated on the left panels. See S1 Table for parameter definitions.
(PDF)

S9 Fig Responses of critical variables during simulated WT and *mPer2^Luc^* rhythm production. WT period = 23.8 h; *mPer2^Luc^* period = 24.1 h. Time dependence of model output variables. The effects on E-box occupation/activation and PER1, PER2, BMAL/CLOCK total concentration as a result of changing each parameter to obtain the altered period in DD. Solid lines = WT model. Dashed lines = mPer2Lucmodel. Time courses are normalized so that maximum E-box activation occurs at time = 0. Common Y-axis labels are indicated on the left panels. See S1 Table for parameter definitions.
(PDF)

S10 Fig Responses of critical variables during simulated WT and *mPer2^Luc^* rhythm production. WT period = 23.8 h; *mPer2^Luc^* period = 24.1 h. Time dependence of model output variables. The effects on E-box occupation/activation and PER1, PER2, BMAL/CLOCK total concentration as a result of changing each parameter to obtain the altered period in DD. Solid lines = WT model. Dashed lines = mPer2Lucmodel. Time courses are normalized so that maximum E-box activation occurs at time = 0. Common Y-axis labels are indicated on the left panels. See S1 Table for parameter definitions.
(PDF)

S11 Fig Responses of critical variables during simulated WT and *mPer2^Luc^* rhythm production. WT period = 23.8 h; *mPer2^Luc^* period = 24.1 h. Time dependence of model output variables. The effects on E-box occupation/activation and PER1, PER2, BMAL/CLOCK total concentration as a result of changing each parameter to obtain the altered period in DD. Solid lines = WT model. Dashed lines = mPer2Lucmodel. Time courses are normalized so that maximum E-box activation occurs at time = 0. Common Y-axis labels are indicated on the left panel. See S1 Table for parameter definitions.
(PDF)

S1 Table Sensitivity of the model to alteration of individual parameters at the baseline period of 23.8 hours. (PDF)
