# Supplementary figures and images for: Targeted modification of the Per2 clock gene alters circadian function in mPer2luciferase (mPer2Luc) mice
Source: PLoS Comput Biol. 2021 May 28;17(5):e1008987. doi: 10.1371/journal.pcbi.1008987 (PMC8191895; doi:10.1371/journal.pcbi.1008987)

DD →

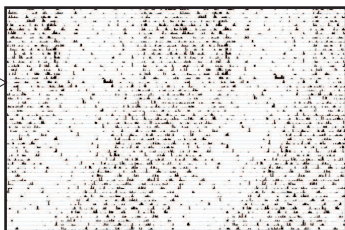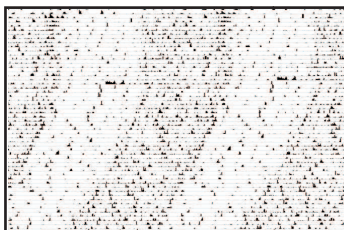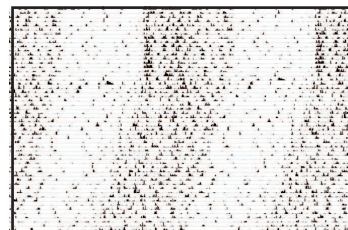

DD →

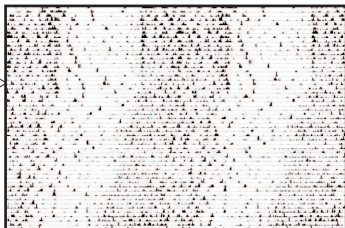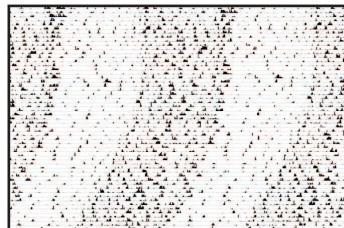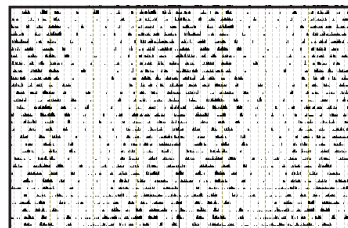

DD →

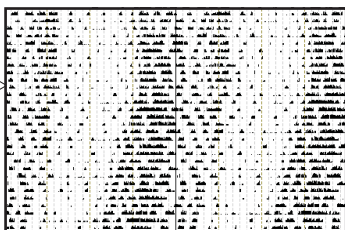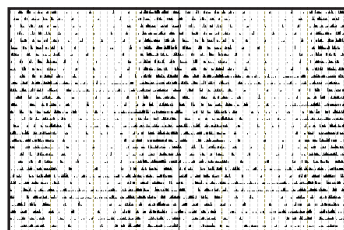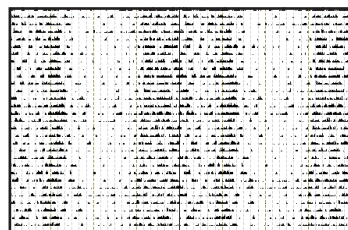

DD →

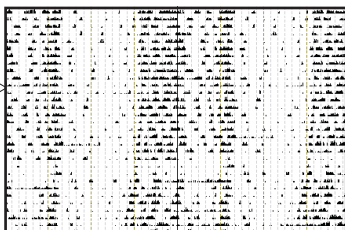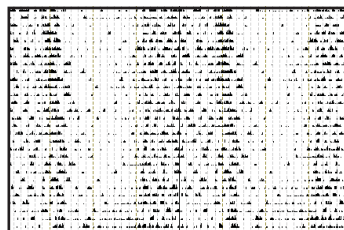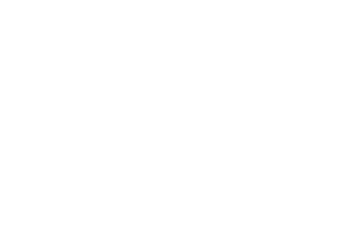

Supplement: S1 Fig — Locomotor activity of wild-type (WT) mice (n = 11) recorded as spontaneous open field activity using infrared activity detectors. Mice were entrained to light/dark cycle LD12:12 (LD) and then released into constant darkness (DD) (arrow). (PDF) [file pcbi.1008987.s001.pdf]

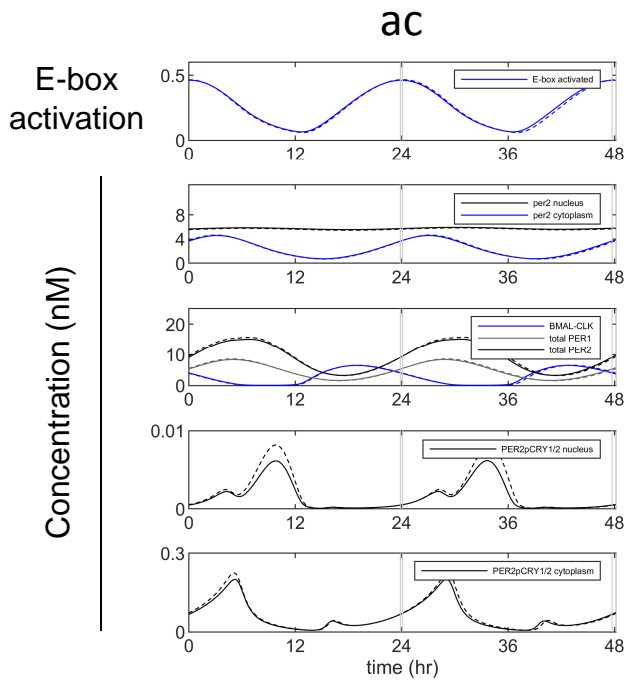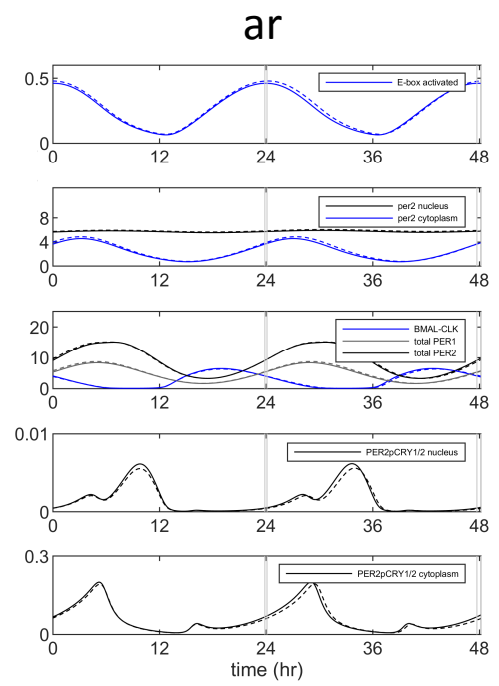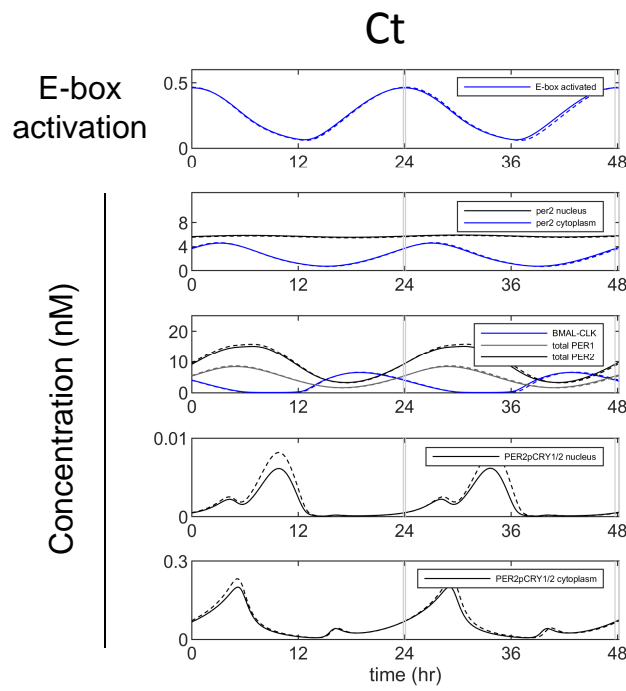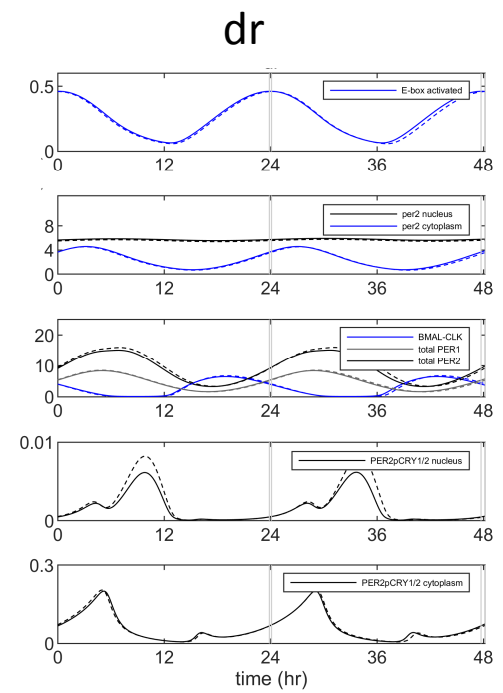

Supplement: S4 Fig — WT period = 23.8 h; mPer2Luc period = 24.1 h. Time dependence of model output variables. The effects on E-box occupation/activation and PER1, PER2, BMAL/CLOCK total concentration as a result of changing each parameter to obtain the altered period in DD. Solid lines = WT model. Dashed lines = mPer2Lucmodel. Time courses are normalized so that maximum E-box activation occurs at time = 0. Common Y-axis labels are indicated on the left panels. See S1 Table for parameter definitions. (PDF) [file pcbi.1008987.s004.pdf]

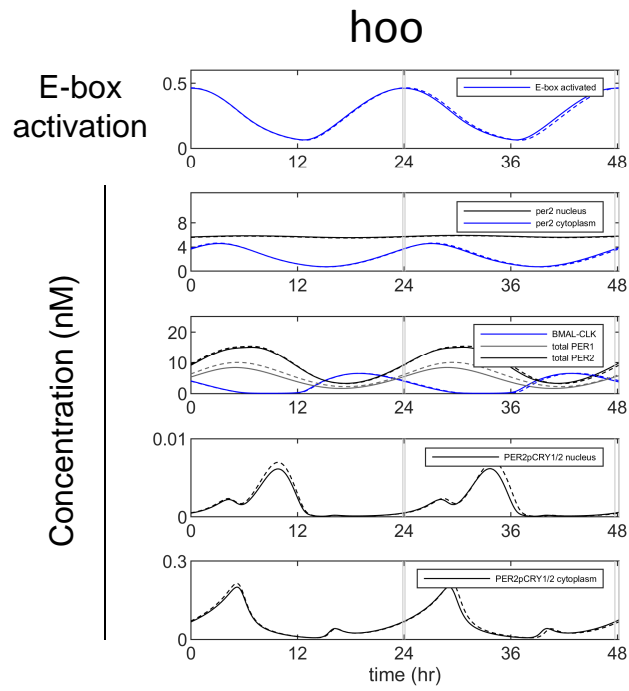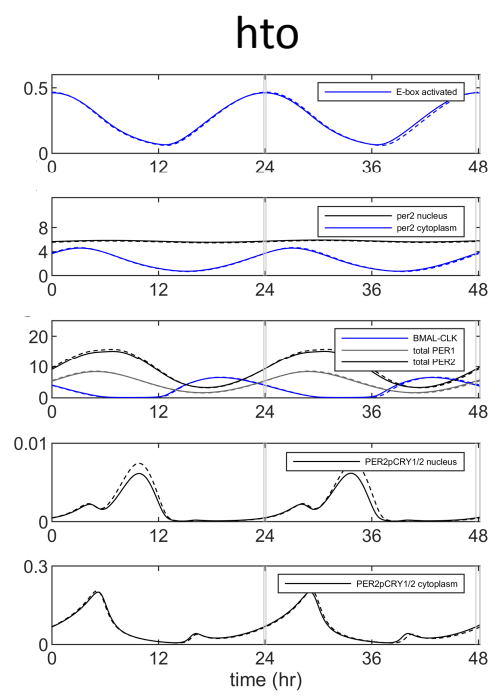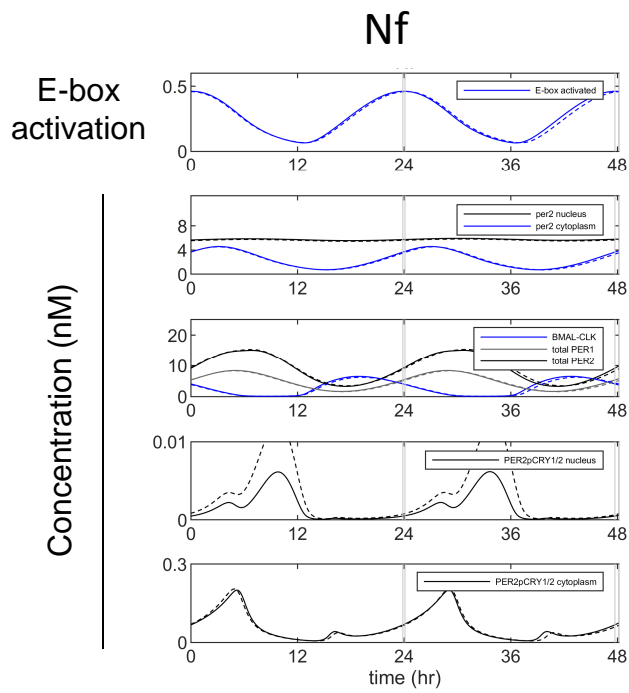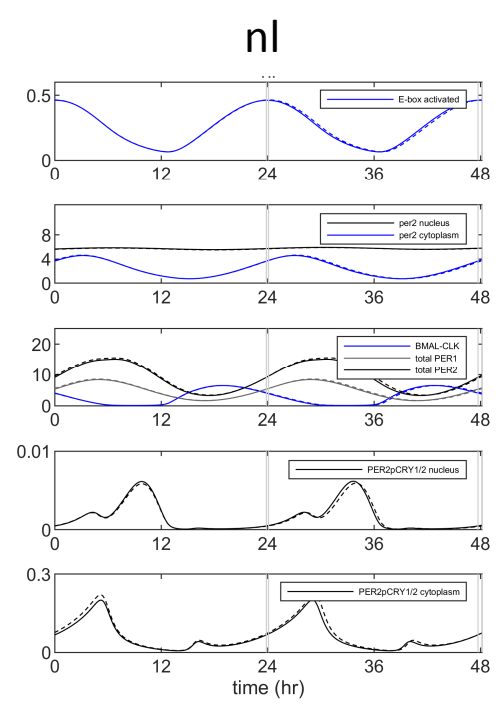

Supplement: S5 Fig — WT period = 23.8 h; mPer2Luc period = 24.1 h. Time dependence of model output variables. The effects on E-box occupation/activation and PER1, PER2, BMAL/CLOCK total concentration as a result of changing each parameter to obtain the altered period in DD. Solid lines = WT model. Dashed lines = mPer2Lucmodel. Time courses are normalized so that maximum E-box activation occurs at time = 0. Common Y-axis labels are indicated on the left panels. See S1 Table for parameter definitions. (PDF) [file pcbi.1008987.s005.pdf]

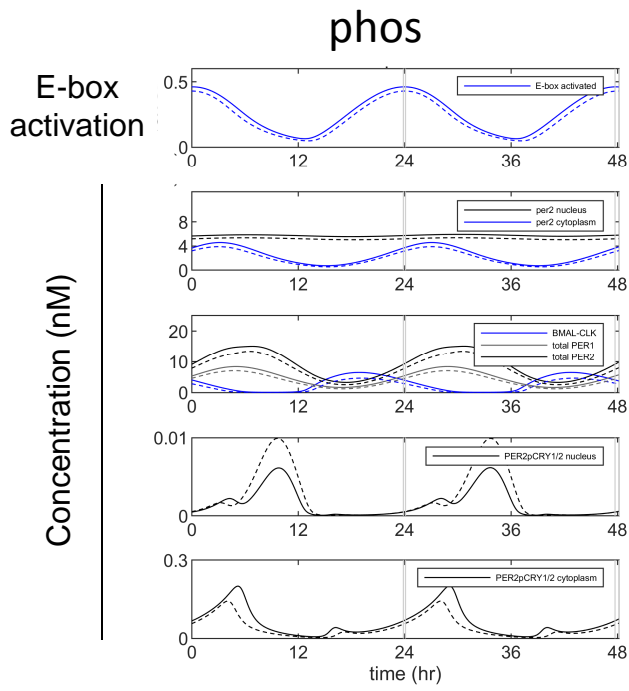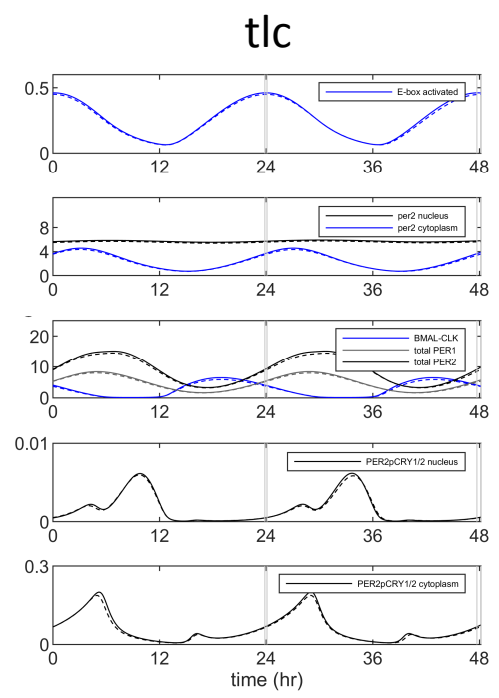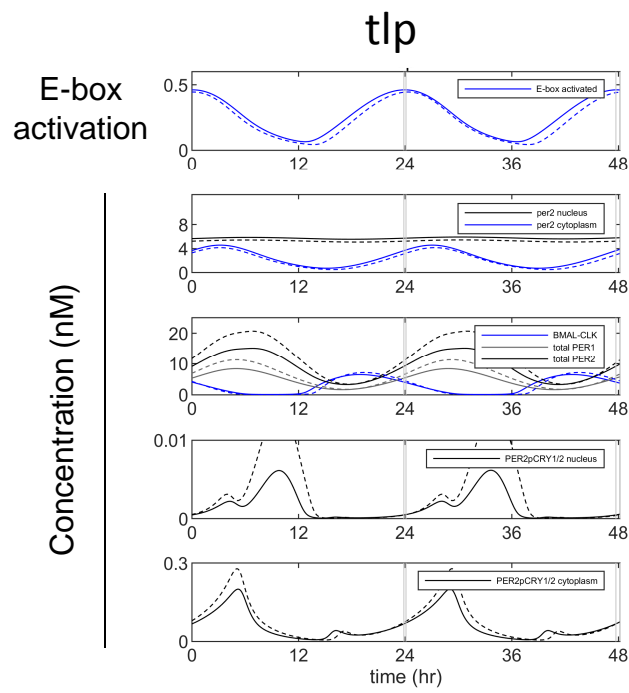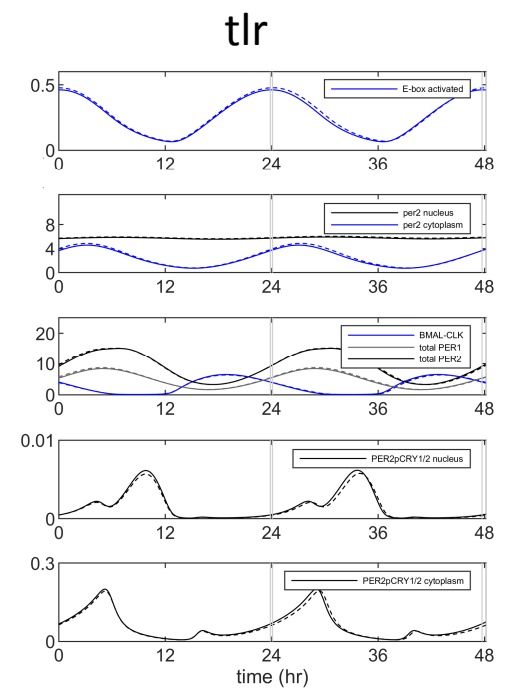

Supplement: S6 Fig — WT period = 23.8 h; mPer2Luc period = 24.1 h. Time dependence of model output variables. The effects on E-box occupation/activation and PER1, PER2, BMAL/CLOCK total concentration as a result of changing each parameter to obtain the altered period in DD. Solid lines = WT model. Dashed lines = mPer2Lucmodel. Time courses are normalized so that maximum E-box activation occurs at time = 0. Common Y-axis labels are indicated on the left panels. See S1 Table for parameter definitions. (PDF) [file pcbi.1008987.s006.pdf]

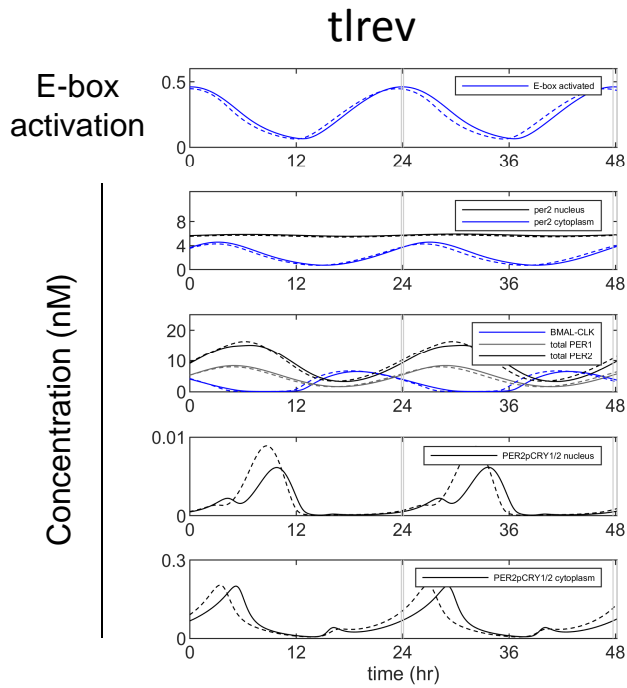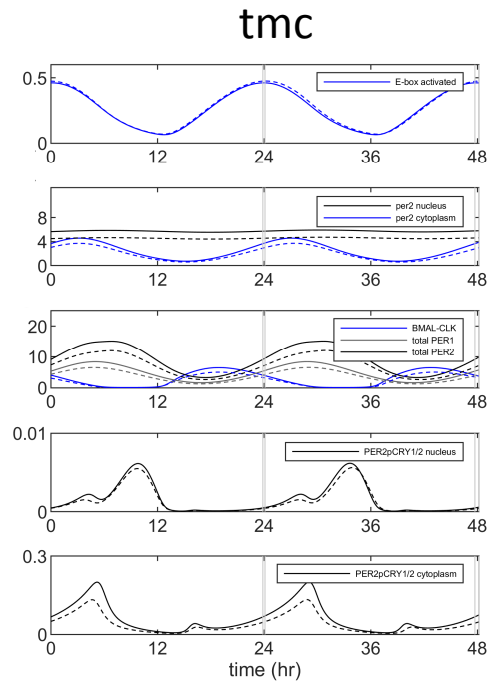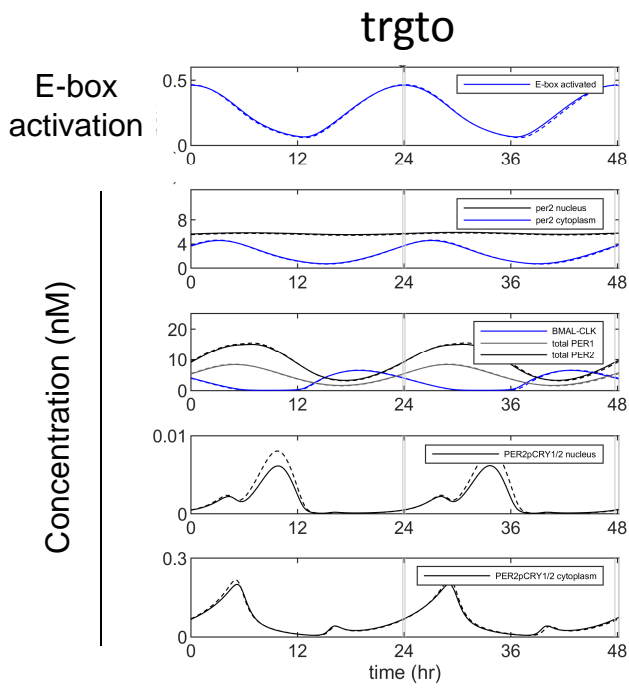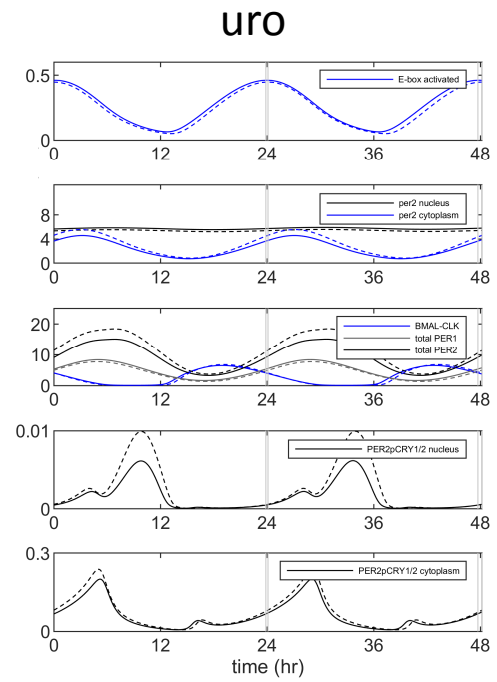

Supplement: S7 Fig — WT period = 23.8 h; mPer2Luc period = 24.1 h. Time dependence of model output variables. The effects on E-box occupation/activation and PER1, PER2, BMAL/CLOCK total concentration as a result of changing each parameter to obtain the altered period in DD. Solid lines = WT model. Dashed lines = mPer2Lucmodel. Time courses are normalized so that maximum E-box activation occurs at time = 0. Common Y-axis labels are indicated on the left panels. See S1 Table for parameter definitions. (PDF) [file pcbi.1008987.s007.pdf]

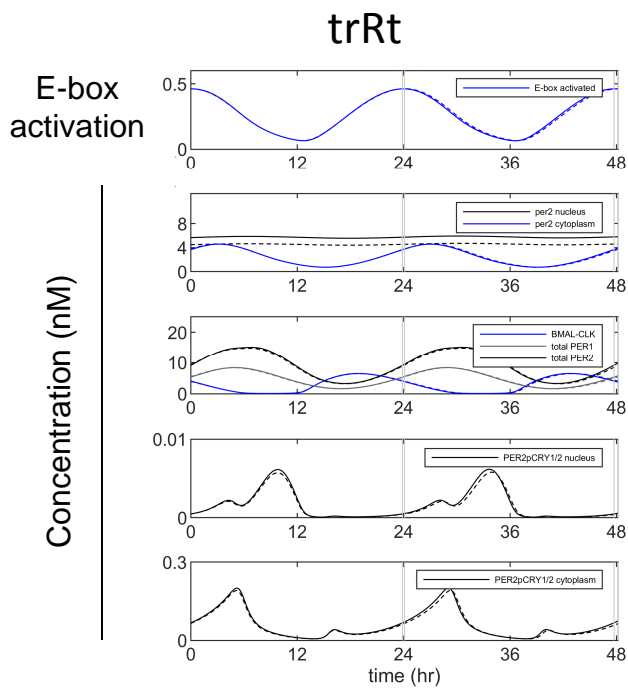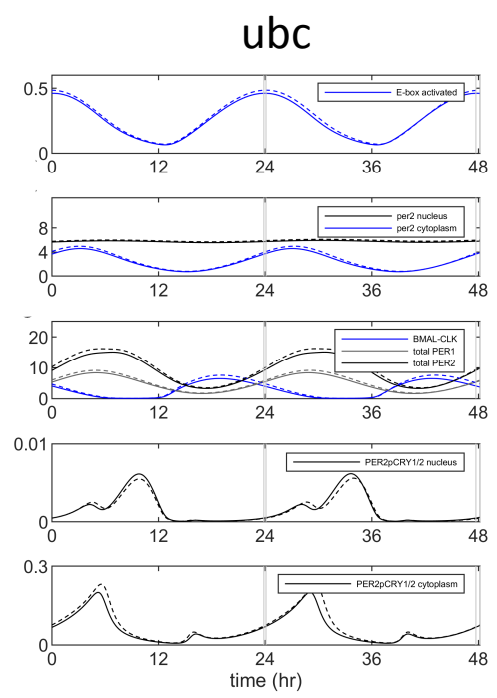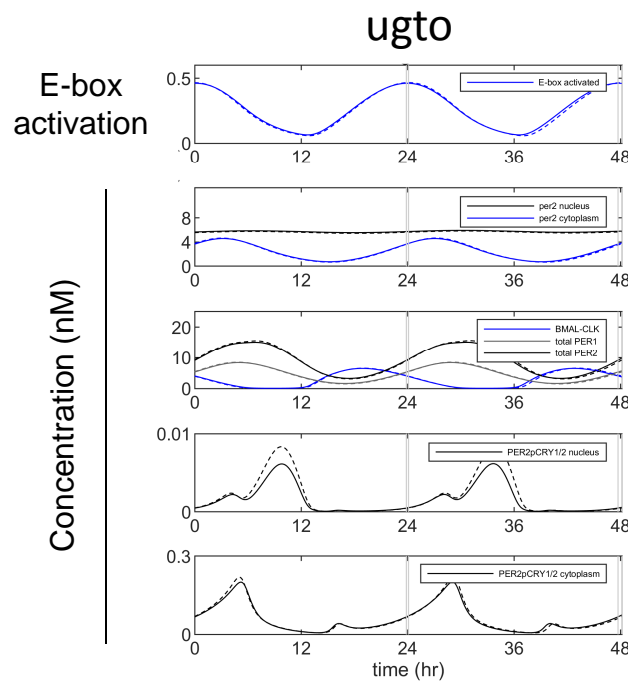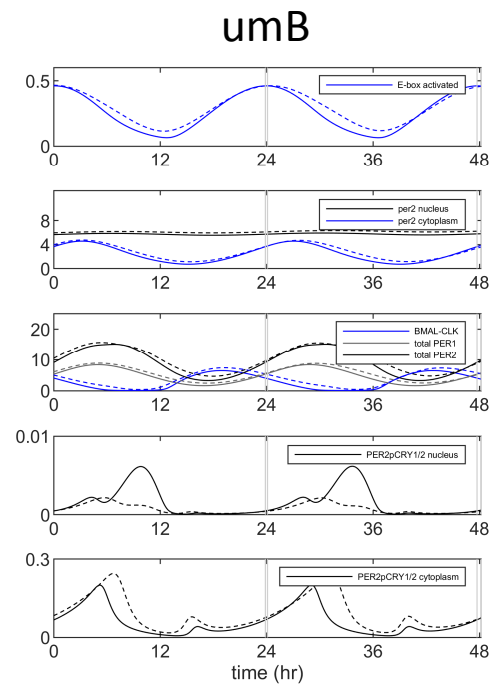

Supplement: S8 Fig — WT period = 23.8 h; mPer2Luc period = 24.1 h. Time dependence of model output variables. The effects on E-box occupation/activation and PER1, PER2, BMAL/CLOCK total concentration as a result of changing each parameter to obtain the altered period in DD. Solid lines = WT model. Dashed lines = mPer2Lucmodel. Time courses are normalized so that maximum E-box activation occurs at time = 0. Common Y-axis labels are indicated on the left panels. See S1 Table for parameter definitions. (PDF) [file pcbi.1008987.s008.pdf]

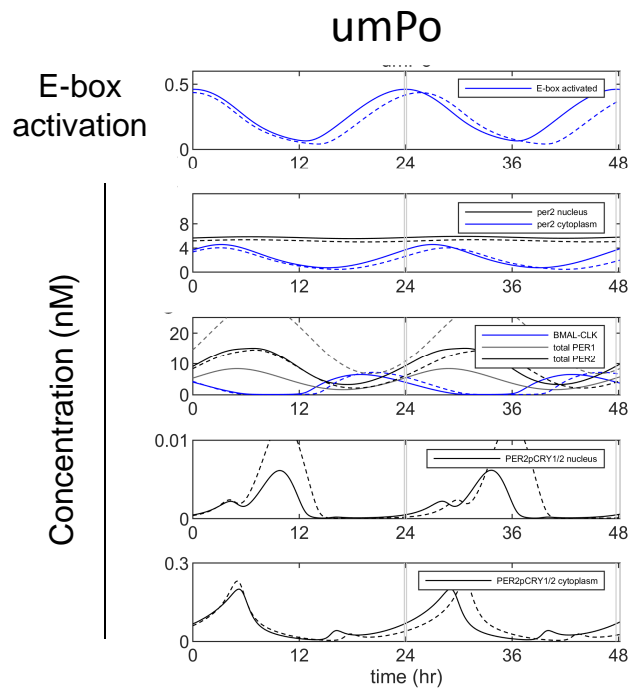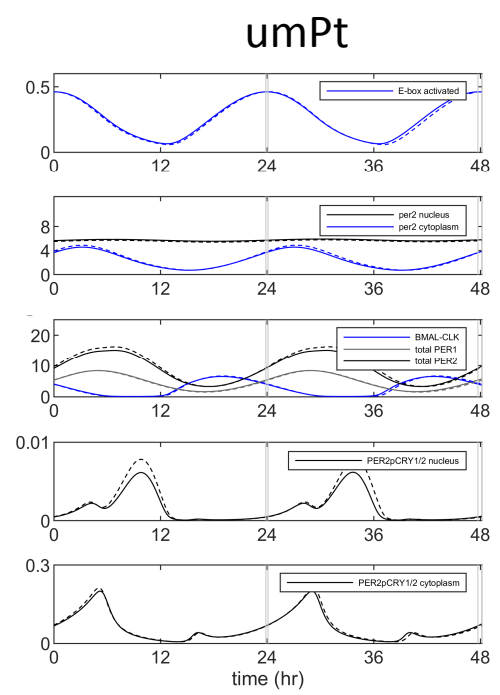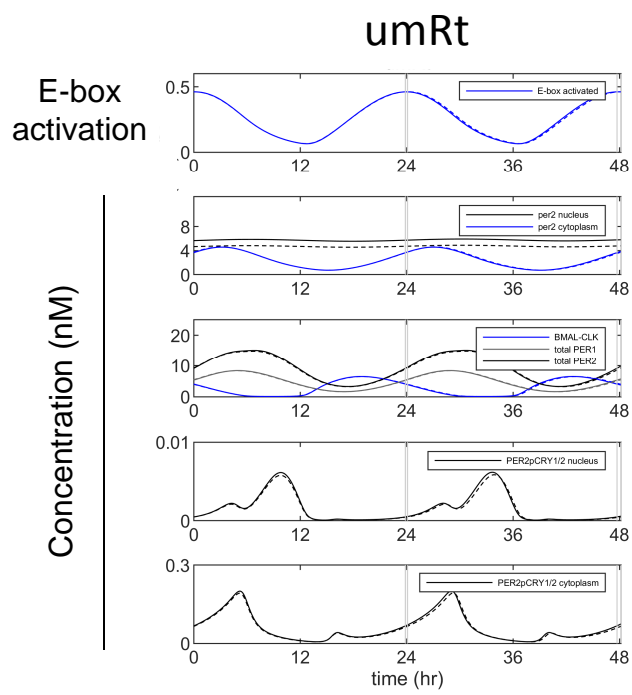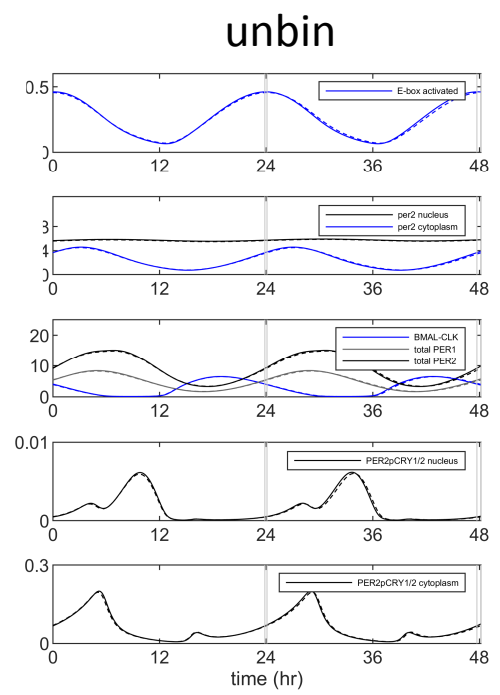

Supplement: S9 Fig — WT period = 23.8 h; mPer2Luc period = 24.1 h. Time dependence of model output variables. The effects on E-box occupation/activation and PER1, PER2, BMAL/CLOCK total concentration as a result of changing each parameter to obtain the altered period in DD. Solid lines = WT model. Dashed lines = mPer2Lucmodel. Time courses are normalized so that maximum E-box activation occurs at time = 0. Common Y-axis labels are indicated on the left panels. See S1 Table for parameter definitions. (PDF) [file pcbi.1008987.s009.pdf]

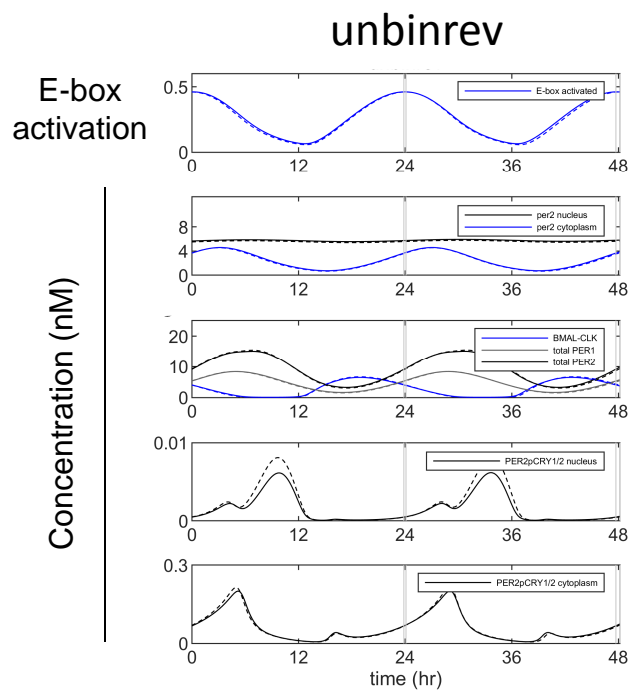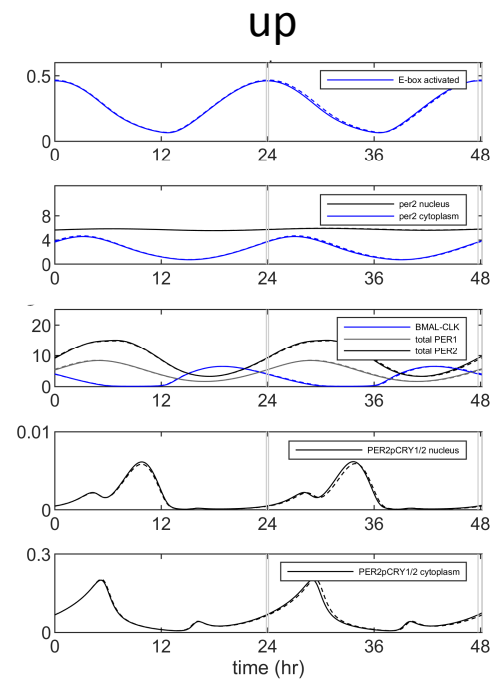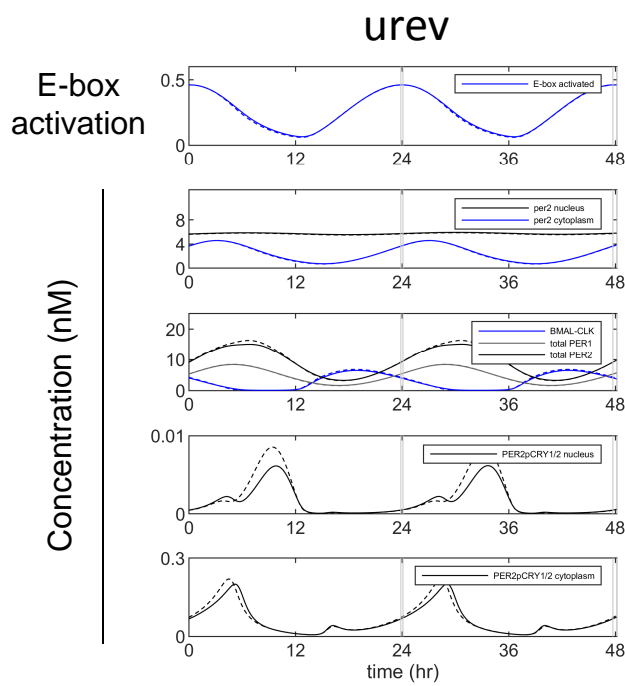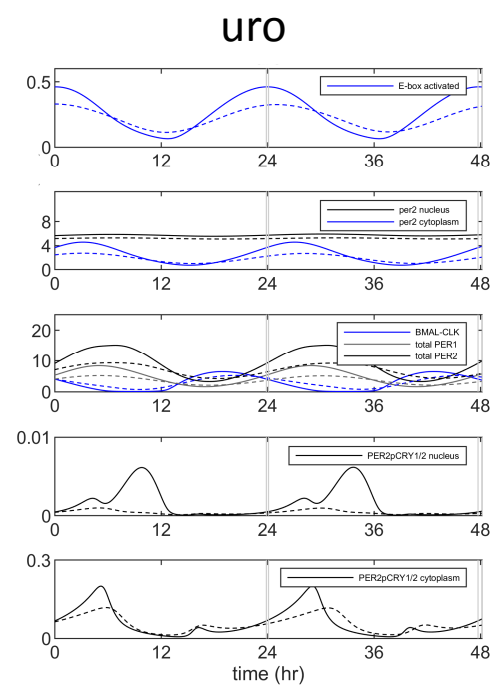

Supplement: S10 Fig — WT period = 23.8 h; mPer2Luc period = 24.1 h. Time dependence of model output variables. The effects on E-box occupation/activation and PER1, PER2, BMAL/CLOCK total concentration as a result of changing each parameter to obtain the altered period in DD. Solid lines = WT model. Dashed lines = mPer2Lucmodel. Time courses are normalized so that maximum E-box activation occurs at time = 0. Common Y-axis labels are indicated on the left panels. See S1 Table for parameter definitions. (PDF) [file pcbi.1008987.s010.pdf]

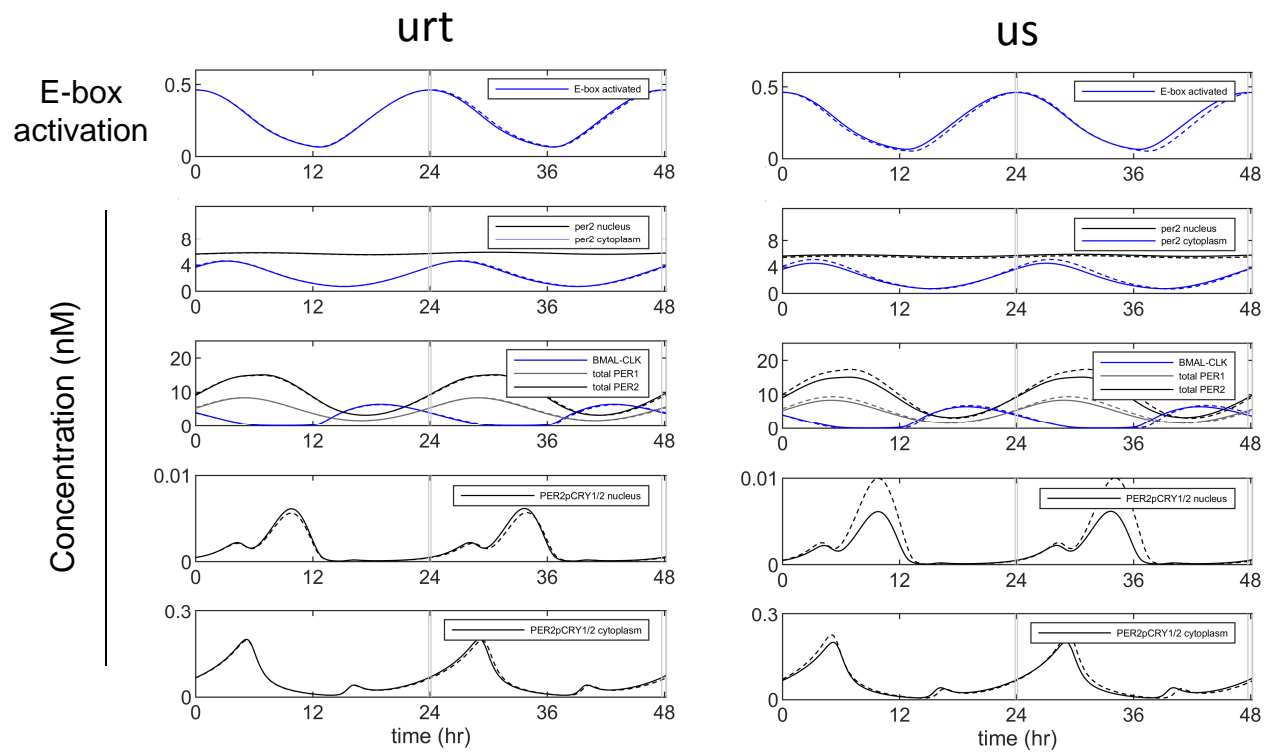

Supplement: S11 Fig — WT period = 23.8 h; mPer2Luc period = 24.1 h. Time dependence of model output variables. The effects on E-box occupation/activation and PER1, PER2, BMAL/CLOCK total concentration as a result of changing each parameter to obtain the altered period in DD. Solid lines = WT model. Dashed lines = mPer2Lucmodel. Time courses are normalized so that maximum E-box activation occurs at time = 0. Common Y-axis labels are indicated on the left panel. See S1 Table for parameter definitions. (PDF) [file pcbi.1008987.s011.pdf]
