# Supplementary material for: Targeted modification of the Per2 clock gene alters circadian function in mPer2luciferase (mPer2Luc) mice
Source: PLoS Comput Biol. 2021 May 28;17(5):e1008987. doi: 10.1371/journal.pcbi.1008987 (PMC8191895; doi:10.1371/journal.pcbi.1008987)

01 Per2(Luc) LD-DD-LL

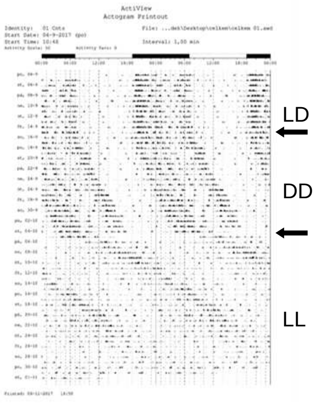

02 Per2(Luc) LD-DD-LL

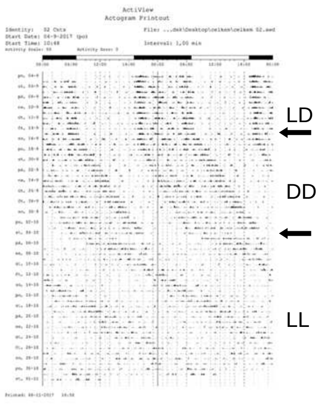

03 Per2(Luc) LD-DD-LL

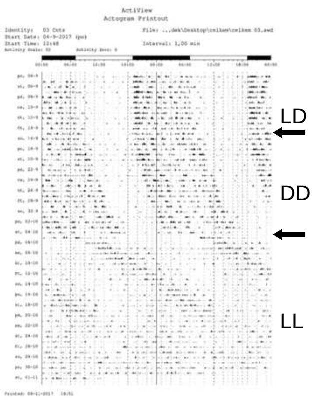

04 Per2(Luc) LD-DD-LL

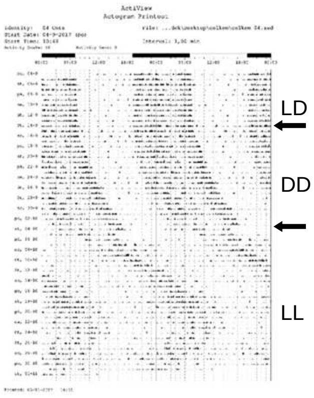

05 Per2(Luc) LD-DD-LL

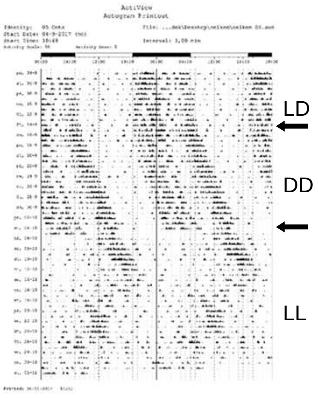

06 Per2(Luc) LD-DD-LL

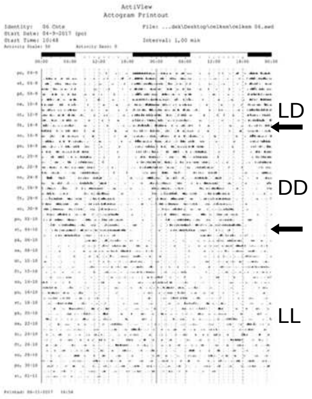

19 WT Per2 LD-DD-LL

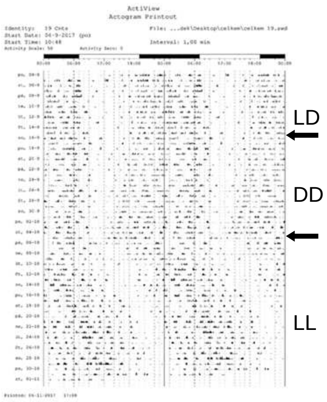

20 WT Per2 LD-DD-LL

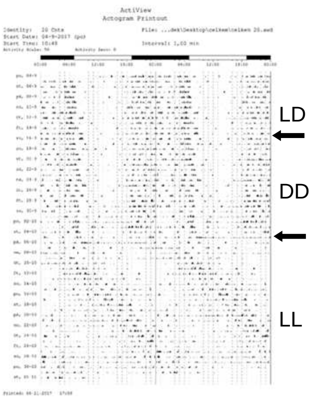

21 WT Per2 LD-DD-LL

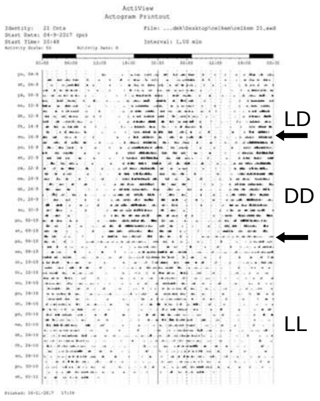

22 WT Per2 LD-DD-LL

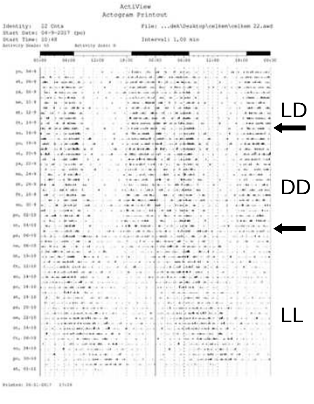

23 WT Per2 LD-DD-LL

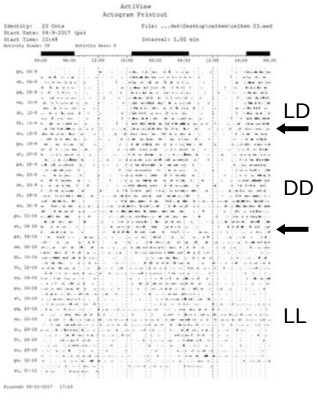

24 WT Per2 LD-DD-LL

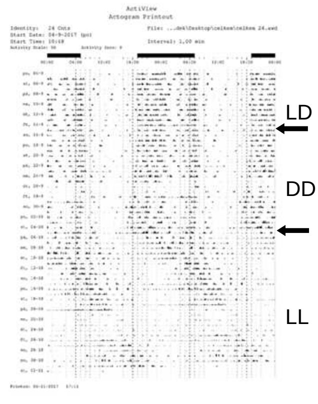

Supplement: S3 Fig — Locomotor activity of wild-type (WT) mice (n = 6) and mPer2Luc/Luc (n = 6) recorded as spontaneous open field activity using infrared activity detectors. Mice were entrained to light/dark cycle LD12:12 (LD), and then released into constant darkness (DD) followed by constant light (LL). (PDF) [file pcbi.1008987.s003.pdf]
