# Supplementary material for: Targeted modification of the Per2 clock gene alters circadian function in mPer2luciferase (mPer2Luc) mice
Source: PLoS Comput Biol. 2021 May 28;17(5):e1008987. doi: 10.1371/journal.pcbi.1008987 (PMC8191895; doi:10.1371/journal.pcbi.1008987)
Supplement: S2 Fig — Locomotor activity of mPer2Luc/Luc mice (n = 16) recorded as spontaneous open field activity using infrared activity detectors. Mice were entrained to light/dark cycle LD12:12 (LD) and then released into constant darkness (DD) (arrow). (PDF) [file pcbi.1008987.s002.pdf]

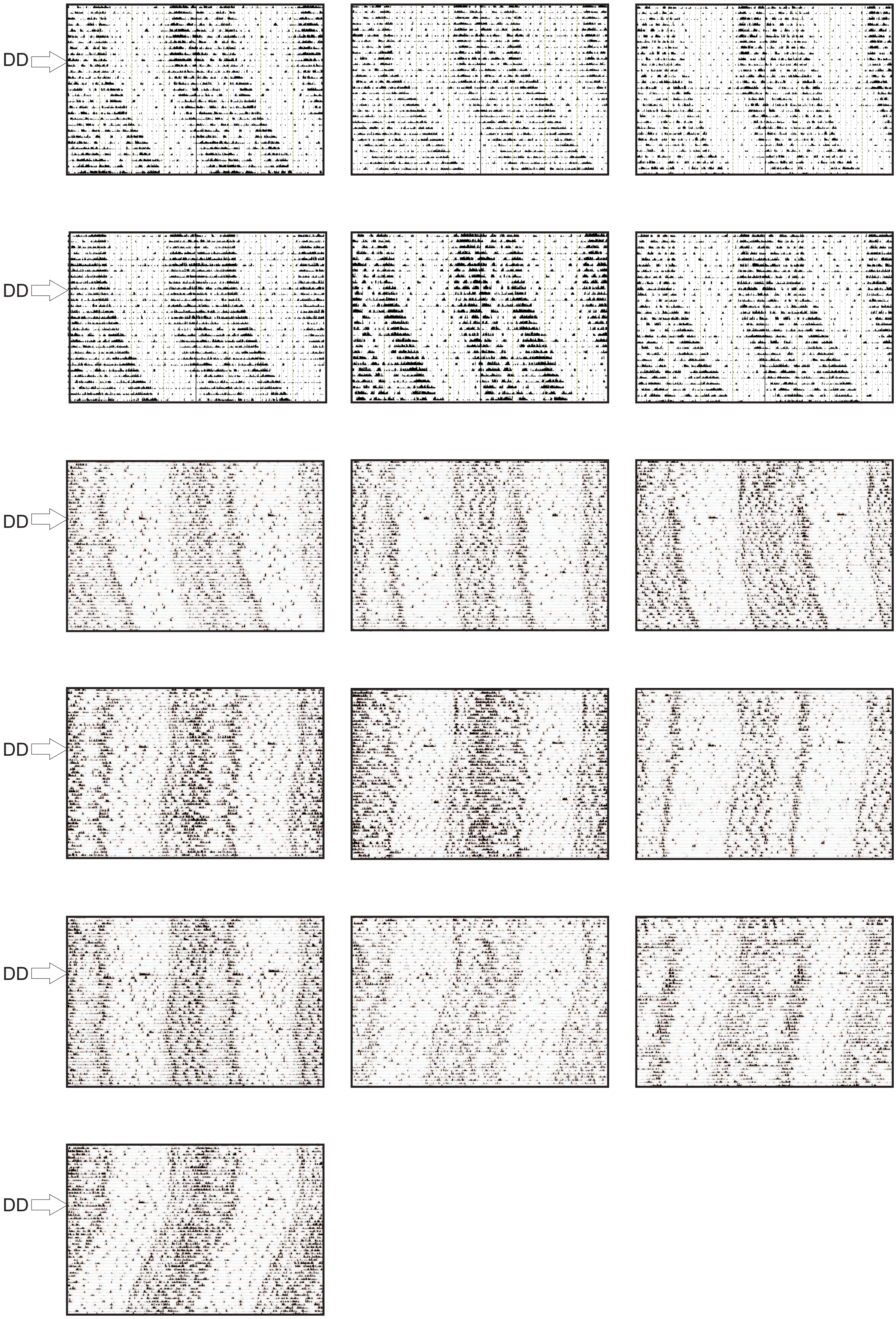
DD
DD
DD
DD
DD
DD
