# Supplementary material for: Targeted modification of the Per2 clock gene alters circadian function in mPer2luciferase (mPer2Luc) mice
Source: PLoS Comput Biol. 2021 May 28;17(5):e1008987. doi: 10.1371/journal.pcbi.1008987 (PMC8191895; doi:10.1371/journal.pcbi.1008987)
Supplement: S1 Table — (PDF) [file pcbi.1008987.s012.pdf]

# Supplementary Table 1

**Sensitivity of the model to alteration of individual parameters at the baseline period of 23.8 hours**

| parameter | description | classification | relative sensitivity of period* |
|---|---|---|---|
| umPt | degradation rate constant for Per2 | Degradation | -0.254 |
| unbin | normalized binding rate constant for BMAL-CLOCK/NPAS2 to Per1/2/Cry1 E-box | Association | -0.223 |
| Ct | total CK1 concentration | Phosphorylation | -0.152 |
| ac | binding rate constant for PER1/2 to CKIε/δ | Phosphorylation | -0.125 |
| hto | CKIε/δ phosphorylation rate constant for PER2 | Phosphorylation | -0.103 |
| ubc | degradation rate constant for BMAL-CLOCK/NPAS2 | Degradation | -0.096 |
| nl | nuclear localization rate constant for proteins bound to PER | Translocation | -0.096 |
| umRt | degradation rate constant for Cry2 | Degradation | 0.089 |
| up | degradation rate constant for CKI phosphorylated PER | Degradation | 0.082 |
| trRt | transcription rate constant for Cry2 | Production | -0.05 |
| tlr | translation rate constant for CRY1 and CRY2 | Production | -0.047 |
| umB | degradation rate constant for Bmal1 | Degradation | -0.047 |
| dr | unbinding rate constant for PER1/2 to CRY1/2 | Association | -0.044 |
| trgto | increase rate of GSK3β activity | Phosphorylation | 0.044 |
| urt | degradation rate constant for Cry2 | Degradation | 0.044 |
| ugto | decrease rate of GSK3β activity | Phosphorylation | -0.043 |
| tlc | translation rate constant for CLOCK | Production | -0.043 |
| ar | binding rate constant for PER1/2 to CRY1/2 | Association | -0.041 |
| tmc | rate constant for folding and nuclear export of Per1/2, Cry1/2, Bmal and Npas2 mRNA | Translocation | -0.037 |
| hoo | CKIε/δ phosphorylation rate constant for PER1 | Phosphorylation | -0.03 |
| uro | degradation rate constant for CRY1 | Degradation | -0.028 |
| Nf | ratio of nuclear to cytoplasmic compartment volume | Translocation | -0.026 |
| phos | phosphorylation rate constant for BMAL-CLOCK/NPAS2 | Phosphorylation | -0.022 |
| trPt | transcription rate constant for of Per2 | Production | 0.021 |
| tlp | translation rate constant for PER1 and PER2 | Production | 0.021 |
| us | clearance rate of cAMP and activated CREB | Degradation | -0.021 |
| umPo | degradation rate constant for Per1 | Degradation | -0.018 |
| unbinrev | normalized unbinding rate constant for REV-ERBs to Cry1/Npas2 RORE | Association | 0.016 |
| tlrev | translation rate constant for REV-ERBs | Production | -0.016 |
| urev | degradation rate constant for unphosphorylated REV-ERBs | Degradation | 0.016 |
| cbin | binding rate constant for BMAL to CLOCK/NPAS2 | Association | -0.015 |
| binrev | normalized binding rate constant for REV-ERBs to Cry1/Npas2 RORE | Association | -0.015 |

| | | | |
|---|---|---|---|
| umRev | degradation rate constant for Rev-Erbs | Degradation | 0.015 |
| uncbin | unbinding rate constant for BMAL to CLOCK/NPAS2 | Association | 0.015 |
| trRev | transcription rate constant for Rev-Erbs | Production | -0.014 |
| uc | degradation rate constant for CLOCK/NPAS2 | Degradation | 0.013 |
| ag | binding rate constant for REV-ERBs to GSK3β | Phosphorylation | 0.012 |
| cbbin | binding rate constant for CRY1/2 to BMAL-CLOCK/NPAS2 in the nucleus | Association | -0.012 |
| tlb | translation rate constant for BMAL | Production | 0.011 |
| Ek | potassium reversal potential | Voltage Dynamics | -0.011 |
| trB | transcription rate constant for Bmal | Production | 0.01 |
| dc | unbinding rate constant for PER1/2 to CKIε/δ | Phosphorylation | 0.01 |
| ts | clearance time constant for shell calcium | Calcium Dynamics | -0.009 |
| lne | nuclear export rate constant for unbound kinases GSK3β and CKI | Translocation | 0.008 |
| trPo | transcription rate constant for Per1 | Production | -0.007 |
| CtrPt | transcription rate of Per2 from CRE activation | Production | 0.007 |
| ks | conversion factor between calcium current and shell calcium concentration | Calcium Dynamics | -0.007 |
| tc | clearance time constant for cytosolic calcium | Calcium Dynamics | 0.007 |
| upu | degradation rate constant for unphosphorylated PER | Degradation | -0.007 |
| dg | unbinding rate constant for PER2/REV-ERBs to GSK3β | Phosphorylation | -0.006 |
| unsbin | rate constant for activated CREB unbinding from CRE site | Coupling | -0.006 |
| bbin | binding rate constant for PER1/2 to BMAL-CLOCK/NPAS2 in the nucleus | Association | -0.006 |
| binrevb | normalized binding rate constant for REV-ERBs to Bmal RORE | Association | -0.006 |
| trRo | transcription rate constant for of Cry1 | Production | 0.005 |
| clk | the strength of the influence of the molecular clock on the electrical activity of SCN neurons | Voltage Dynamics | 0.005 |
| uncbbin | unbinding rate constant for CRY1/2 to BMAL-CLOCK/NPAS2 in the nucleus | Association | 0.005 |
| unbinrevb | normalized unbinding rate constant for REV-ERBs to Bmal RORE | Association | 0.005 |
| unbinc | normalized unbinding rate constant for BMAL-CLOCK/NPAS2 to Cry2 E-box | Association | -0.004 |
| umNp | degradation rate constant for Npas2 | Degradation | 0.004 |
| Eca | calcium reversal potential | Voltage Dynamics | -0.004 |
| sbin | rate constant for activated CREB binding to CRE site on DNA | Coupling | 0.003 |
| kc | conversion factor between calcium current and cytosolic calcium concentration | Calcium Dynamics | 0.003 |
| nlrev | nuclear localization rate constant for REV-ERBs as well as GSK3β if bound | Translocation | -0.003 |
| Ena | sodium reversal potential | Voltage Dynamics | 0.003 |
| K2 | sigmoid parameter for inactivation of L-type calcium channels | Calcium Dynamics | 0.003 |
| uprev | degradation rate constant for GSK3β phosphorylated REV-ERBs | Degradation | 0.002 |
| ub | degradation rate constant for Bmal | Degradation | -0.002 |
| umRo | degradation rate constant for Cry1 | Degradation | -0.002 |
| bs | basal release rate of shell calcium | Calcium Dynamics | -0.002 |

| | | | |
|---|---|---|---|
| gna | maximum sodium conductance | Voltage Dynamics | -0.002 |
| taurl | time constant of L-type calcium channels | Calcium Dynamics | -0.002 |
| gcanl | maximum conductance of non-L-type calcium channels | Voltage Dynamics | -0.002 |
| nlbc | nuclear localization rate constant for BMAL-CLOCK/NPAS2 | Translocation | -0.002 |
| tmcrev | rate constant for folding and nuclear export of Rev-Erbs mRNA | Translocation | -0.001 |
| trNp | transcription rate constant for Npas2 | Production | -0.001 |
| K1 | sigmoid parameter for inactivation of L-type calcium channels | Calcium Dynamics | 0.001 |
| uv | degradation rate of VIP | Degradation | -0.001 |
| CtrPo | transcription rate of Per1 from CRE activation | Production | -0.001 |
| uncvbin | rate constant for CRY1/CRY2 unbinding from VPAC2R | Coupling | -0.001 |
| gk | maximum potassium conductance | Voltage Dynamics | 0.001 |
| unbbin | unbinding rate constant for PER1/2 to BMAL-CLOCK/NPAS2 in the nucleus | Association | -0.001 |
| cvbin | rate constant for CRY1/CRY2 binding to VPAC2R | Coupling | -0.001 |
| vbin | rate constant for VIP binding to VPAC2R | Coupling | 0.001 |
| unbinr | normalized unbinding rate constant for BMAL-CLOCK/NPAS2 to Rev-erbs E-box | Association | -0.001 |
| Vt | VPAC2R expression level | Production | -0.001 |
| binc | normalized binding rate constant for BMAL-CLOCK/NPAS2 to Cry2 E-box | Association | 0.001 |
| tlnp | translation rate constant for NPAS2 | Production | -0.001 |
| ne | nuclear export rate constant for protein bound to PER | Translocation | -0.001 |
| gcal | maximum conductance of L-type calcium channels | Voltage Dynamics | 0.001 |
| nerev | nuclear export rate constant for REV-ERBs as well as GSK3β if bound | Translocation | -0.001 |
| Gt | total GSK3β concentration | Phosphorylation | 0.001 |
| agp | binding rate constant for PER2 to GSK3β | Phosphorylation | 0 |
| c | membrane capacitance | Voltage Dynamics | 0 |
| binr | normalized binding rate constant for BMAL-CLOCK/NPAS2 to Rev-erbs E-box | Association | 0 |
| vs | cAMP production rate (when VPAC2R bound by VIP & not inhibited by CRY) (= activation rate of CREB by cAMP) | Coupling | 0 |
| bin | normalized binding rate constant for BMAL-CLOCK/NPAS2 to Per1/2/Cry1 E-box | Association | 0 |
| taurnl | time constant of non-L-type calcium channels | Calcium Dynamics | 0 |
| unvbin | rate constant for VIP unbinding from VPAC2R | Coupling | 0 |
| bc | basal release rate of cytosolic calcium | Calcium Dynamics | 0 |
| gnaleak | sodium leak channel conductance | Voltage Dynamics | 0 |
| vpr | VIP release rate | Association | 0 |
| gkca | maximal conductance of calcium-dependent potassium channels | Voltage Dynamics | 0 |
| gkleak | potassium leak channel conductance | Voltage Dynamics | 0 |
| aR | rate of GABA synapic channel opening | Voltage Dynamics | 0 |
| aD | rate of GABA synapic channel closing | Voltage Dynamics | 0 |

Supplementary Table 1

| | | | |
|---|---|---|---|
| Tmax | maximum released GABA neurotransmitter concentration | Voltage Dynamics | 0 |
| VT | GABA synapse release threshold | Voltage Dynamics | 0 |
| Kp | GABA synapse release sensitivity | Voltage Dynamics | 0 |
| Egaba | GABA synaptic current reversal potential | Voltage Dynamics | 0 |
| ggaba | maximum conductance of the GABA synaptic current | Voltage Dynamics | 0 |
| Iglut | strength of the current caused by glutamate input (irrelevant in DD) | Voltage Dynamics | 0 |

**** Positive values occur when increasing the parameter value increases period.***

Highlighted parameters increase period from wild type to mPer2(Luc) with a <25% change from baseline
